# Indispensable epigenetic control of thymic epithelial cell development and function by polycomb repressive complex 2

**DOI:** 10.1038/s41467-021-24158-w

**Published:** 2021-06-24

**Authors:** Thomas Barthlott, Adam E. Handel, Hong Ying Teh, Rushika C. Wirasinha, Katrin Hafen, Saulius Žuklys, Benoit Roch, Stuart H. Orkin, Jean-Pierre de Villartay, Stephen R. Daley, Georg A. Holländer

**Affiliations:** 1 Department of Biomedicine and University Children’s Hospital of Basel, University of Basel, Basel, Switzerland; 2 Nuffield Department of Clinical Neurosciences, University of Oxford, Oxford, UK; 3 Infection and Immunity Program, Monash Biomedicine Discovery Institute and Department of Biochemistry and Molecular Biology, Monash University, Melbourne, VIC Australia; 4 Genome Dynamics in the Immune System Laboratory, INSERM UMR 1163, Université de Paris, Imagine Institute, Paris, France; 5 Department of Pediatric Oncology, Dana-Farber Cancer Institute and Division of Hematology/Oncology, Boston Children’s Hospital, Harvard Stem Cell Institute, Harvard Medical School, and Howard Hughes Medical Institute, Boston, MA USA; 6 Department of Paediatrics and the Weatherall Institute of Molecular Medicine, University of Oxford, Oxford, UK; 7 Present Address: School of Health and Biomedical Sciences, Queensland University of Technology, Brisbane, QLD Australia

**Keywords:** Clonal selection, Autoimmunity, Epigenetics in immune cells, Thymus

## Abstract

Thymic T cell development and T cell receptor repertoire selection are dependent on essential molecular cues provided by thymic epithelial cells (TEC). TEC development and function are regulated by their epigenetic landscape, in which the repressive H3K27me3 epigenetic marks are catalyzed by polycomb repressive complex 2 (PRC2). Here we show that a TEC-targeted deficiency of PRC2 function results in a hypoplastic thymus with reduced ability to express antigens and select a normal repertoire of T cells. The absence of PRC2 activity reveals a transcriptomically distinct medullary TEC lineage that incompletely off-sets the shortage of canonically-derived medullary TEC whereas cortical TEC numbers remain unchanged. This alternative TEC development is associated with the generation of reduced TCR diversity. Hence, normal PRC2 activity and placement of H3K27me3 marks are required for TEC lineage differentiation and function and, in their absence, the thymus is unable to compensate for the loss of a normal TEC scaffold.

## Abstract (web summary)

Development of the T cells requires functions from thymic epithelial cells, whose development and function are epigenetically regulated. Here the authors show that inactivation of the polycomb repressive complex 2 (PRC2) alters the H3K27me3 configuration, reduces TEC functions, reveals a specific TEC subset, and hampers T cell development.

## Introduction

The controlled balance between cell proliferation and differentiation is essential to maintain a normal thymic microenvironment to promote the development and selection of naive T cells with a repertoire purged of vital “self” specificities but prepared to react to injurious “non-self”. Thymic epithelial cells (TECs), which can be categorized based on specific molecular, structural and functional characteristics into separate cortical (cTECs) and medullary (mTECs) lineages, are essential for this competence^1^. Both TEC lineages derive from a common epithelial precursor of endodermal origin^2,3^ that expresses a range of cTEC-specific markers including the proteasome component β5t encoded by *Psmb11*^4–7^.

cTECs induce the commitment of blood-borne precursor cells to a T cell fate, foster their subsequent growth and initial maturation, and select immature thymocytes that express a T cell antigen receptor (TCR) with sufficient affinity for self-peptides presented by major histocompatibility complexes (pMHC). In contrast, mTECs promote the terminal differentiation of thymocytes, which includes the establishment of immunological tolerance to tissue-restricted antigens (TRAs) via two complementary mechanisms; a recessive activity of deleting thymocytes expressing a TCR with high affinity for these antigens, and a dominant mechanism of clonal diversion that generates thymic regulatory T cells^8^. In addition, thymus immigrating dendritic cells (DCs) and B cells provide peripheral antigens for central tolerance induction^9–11^.

The ectopic representation of a significant number of TRAs by a subpopulation of phenotypically mature mTECs is in part dependent on the expression of the transcriptional facilitator autoimmune regulator (AIRE)^12^, which acts independently of lineage-specific transcription factors^13^. Individual mTECs express AIRE-dependent TRAs to an extent significantly lower than that of corresponding peripheral tissues^14,15^ and in co-expression patterns that change with mTEC maturation^16–19^. AIRE limits the amplitude of TRA gene expression by repressing chromatin accessibility at distal cis-regulatory sequences^20^. These regions, ostensibly placed within facultative heterochromatin, are enriched in the repressive histone mark, H3K27me3, and depleted for histone modifications permissive for conventional gene transcription^8^.

H3K27me3 is a posttranslational modification catalysed by polycomb repressive complex 2 (PRC2) that depends for its function on four core factors, including EED (embryonic ectoderm development), which is critical for physically binding to H3K27me3, and both EZH1 and EZH2 (enhancer of zeste homologue), which mediate the trimethylation of H3K27^21,22^. Compromised gene repression due to a loss of PRC2 activity^23^ impairs tissue specification and maintenance, and consequently results in early embryonic lethality^24^.

To establish a specific role for PRC2 in TEC development and function, we analyse mice with a loss of this complex targeted to TEC precursors and occurring after the formation of the thymus anlage. Absent PRC2 activity in TEC leads to an impairment in canonical TEC development and function whilst in parallel an alternative mTEC differentiation pathway emerges. These abnormalities are associated with reduced TCR diversity in the CD4^+^ and CD8^+^ naive T cell pools, yet negative selection of self-reactive TCR specificities remained intact. Taken together, we conclude that PRC2 activity is essential for TEC lineage specification and function.

## Results

### Loss of EED in TEC differentially disrupts lineage development

**Fig. 1:**
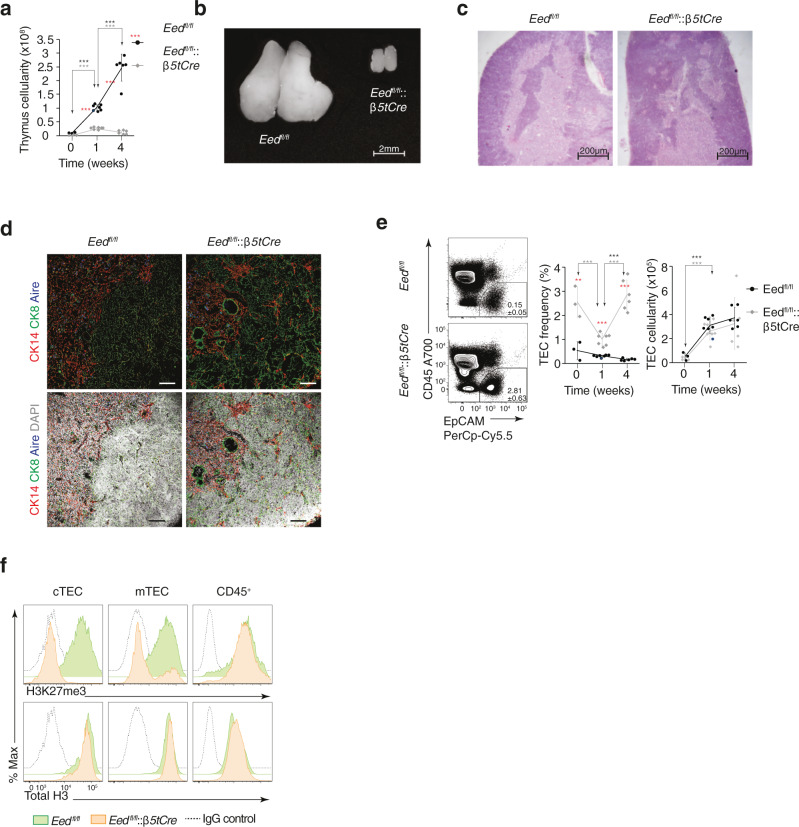
Thymus phenotype of *Eed*^fl/fl^::*β5tCre* mice. **a** Absolute thymus cellularity of mutant (grey diamonds) and control mice (black circles) at the indicated postnatal ages (week 0 relates to newborn). **b** Macroscopic and **c** microscopic comparison of thymic lobes isolated from 4-week-old *Eed*^fl/fl^:*:β5tCre* and control *Eed*^fl/fl^ mice. Scale bars represent 2 mm and 200 µm, respectively. Tissue sections were stained with haematoxylin and eosin. **d** Immunohistology of thymus sections prepared from 4-week-old *Eed*^fl/fl^ and *Eed*^fl/fl^::*β5tCre* mice and stained for cytokeratin (CK) 8 (green, a cTEC marker), CK14 (red, an mTEC marker), AIRE (blue) and DAPI (grey; only in lower panels). Scale bars represent 50 µm. **e** Relative frequency and absolute cellularity of TEC isolated from *Eed*^fl/fl^:*:β5tCre* (grey diamonds) and control *Eed*^fl/fl^ mice (black circles) at indicated ages. Contour plots show representative flow cytometric analyses of thymic single-cell suspensions of 4-week-old mice, the gating for TEC and their relative frequencies. **f** Detection of H3K27me3 marks and histone H3 protein in mTEC, cTEC and CD45^+^ cells isolated from *Eed*^fl/fl^::*β5tCre* and *Eed*^fl/fl^ mice at 4 weeks of age. Isotype control stains for *Eed*^fl/fl^ mice are indicated as IgG control. Data in graphs (**a**, **e**) represent mean and standard deviations (SD) and are pooled from independent experiments: week 0: *n* = 3, week 1: *n* = 7, week 4: *n* = 6; ^*^p < 0.05, ^**^p < 0.01, ^***^p < 0.001 (two-tailed unpaired Student’s *t* test). Asterisks in red indicate comparisons between *Eed*^fl/fl^::*β5tCre* and *Eed*^fl/fl^ mice, whereas black and grey asterisks compare results from identical mouse strains at different time points. Data in (**b**–**d**, **f**) are representative of two independent experiments with *n* = 3 mice each per strain investigated. *n* = biologically independent replicates per group. The gating for thymic epithelia is displayed in Supplementary Fig. 1. Source data including exact statistical test values are provided in the Source Data file.

To generate mice with a loss of PRC2 activity in all thymic epithelia at a time after initiation of a thymus anlage, we interbred animals harbouring a conditional *Eed* allele to mice in which the Cre recombinase is expressed under the control of *Psmb11* regulatory elements^25,26^. The resultant *Eed*^fl/fl^*::β5tCre* mice displayed a small thymus with a reduced total cellularity, a normal corticomedullary segregation and a number of small, cell-free cysts surrounded by cytokeratin 8-positive epithelia (Fig. 1a–d). However, the total number of TEC recovered from these mice was similar to that of wild-type and *Eed*^fl/fl^ mice (Fig. 1e). EED deletion resulted in the expected depletion of H3K27me3 marks in all cTECs and the majority of mTECs isolated from *Eed*^fl/fl^*::β5tCre* mice, whereas H3 histone abundance remained unchanged (Fig. 1f and see below; gating strategy in Supplementary Fig. 1).

**Fig. 2:**
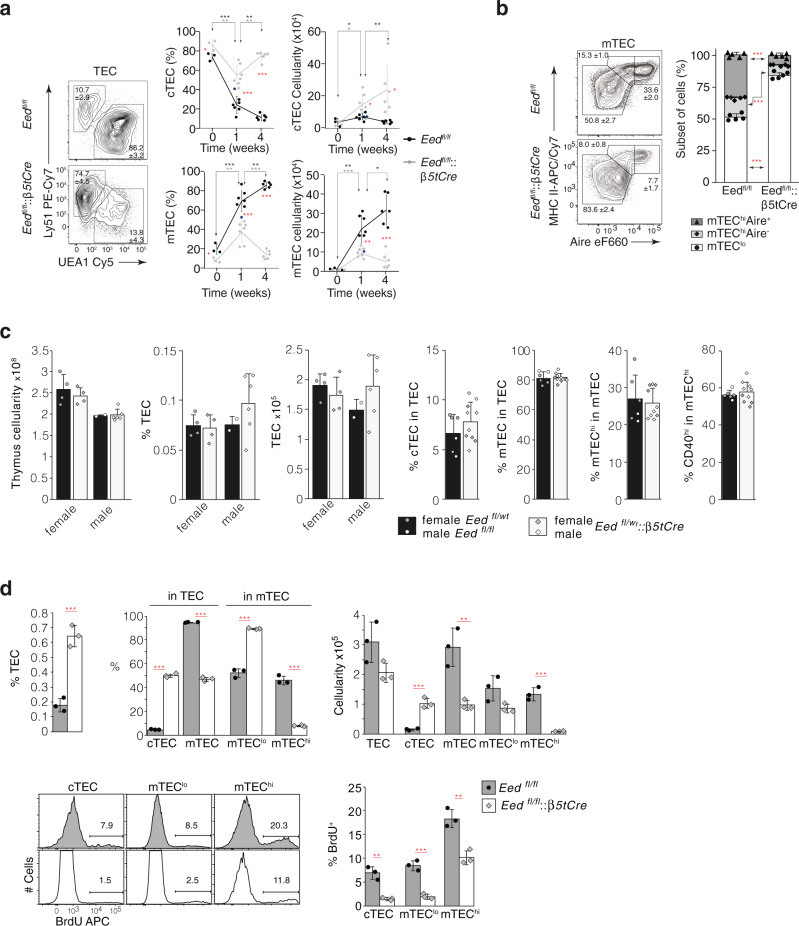
TEC phenotype of *Eed*^fl/fl^::*β5tCre* mice. **a** Quantification of cortical (CD45^−^EpCAM^+^Ly51^+^UEA1^−^) and medullary (CD45^−^EpCAM^+^Ly51^−^UEA1^+^) TEC subpopulations isolated from mice with the indicated genotype. Representative contour plots are from 4-week-old mice, frequencies and absolute cell numbers of cTEC and mTEC are from mice with the indicated genotype (*Eed*^fl/fl^:*:β5tCre*: grey diamonds, *Eed*^fl/fl^ mice: black circles) at ages shown (week 0: *n* = 3, week 1: *n* = 7, week 4: *n* = 6). **b** Flow cytometric analysis of mTEC from 4-week-old mice for MHCII and AIRE expression. Representative contour plots and bar graphs displaying the frequencies of individual subpopulations (mTEC:^lo^ white bars black circles, mTEC^hi^AIRE:^−^ light grey bars black diamonds, mTEC^hi^ AIRE:^+^ grey bars black triangles) for each of the indicated genotypes (*n* = 5). **c** Quantification of total and TEC cellularity and determination of TEC subset distributions in *Eed*^fl/wt^:*:β5tCre* and control mice. Data are pooled from two independent experiments with female (*n* = 4 *Eed*:^fl/wt^:*β5tCre*: light grey bars with grey diamonds, *n* = 4 *Eed*:^fl/wt^ black bars with grey circles) and male mice (*n* = 6 *Eed*^fl/wt^::*β5tCre*: light grey bars with white diamonds, *n* = 2 *Eed*:^fl/fl^ black bars with white circles) at 4–6 weeks of age. Cellularities are displayed for each sex separately, TEC subset frequencies are pooled. **d** TEC subset frequencies, cellularities and proliferation in 4-week-old *Eed*^fl/fl^::*β5tCre* (*n* = 3, white bars with grey diamonds) and *Eed*^fl/fl^ mice (*n* = 3, grey bars with black circles). Representative histograms showing BrdU incorporation and frequencies of BrdU-positive cTEC and mTEC subsets. Data in graphs represent mean and standard deviations (SD) and are pooled from independent experiments (**a**, **c**) or display one experiment representative of two independent experiments (**b**, **d**). Contour plots (**a**, **b**) and histograms (**d**) are representative of data in bar graphs. The gating for thymic epithelia is displayed in Supplementary Fig. 1. *n* = biologically independent replicates per group. ^*^p < 0.05, ^**^p < 0.01, ^***^p < 0.001 (two-tailed unpaired Student’s *t* test). Asterisks in red indicate comparisons between *Eed*^fl/fl^:*:β5tCre* and *Eed*^fl/fl^ mice, whereas black and grey asterisks compare results from identical mouse strains at different time points. Source data including exact statistical test values are provided in the Source Data file.

*Eed*^fl/fl^*::β5tCre* mice had a higher frequency and cellularity of cTECs but a lower rate and reduced cell number of mTECs (Fig. 2a). The difference was present at birth and steadily increased with age. Noticeably, cTEC cellularity did not diminish in adolescent *Eed*^fl/fl^*::β5tCre* mice contrary to controls. Moreover, the maturation of mTEC was compromised in *Eed:*^fl/fl^*:β5tCre* mice with fewer immature epithelia (MHCII^lo^, AIRE^−^, designated mTEC^lo^) attaining a mature (MHC^hi^; mTEC^hi^) cell stage, either positive or negative for the expression of AIRE (Fig. 2b). Heterozygosity for a loss of *Eed* did not compromise thymus cellularity nor affect TEC numbers or subset composition (Fig. 2c). The proliferation rates of both cTEC and mTEC were significantly reduced in *Eed*^fl/fl^*::β5tCre* mice (Fig. 1h and Supplementary Fig. 1) rendering it an unlikely explanation for the disparity in cTEC:mTEC ratios. Thus, the loss of PRC2 activity (consequent to an absence of either EED or EZH1/2; Supplementary Fig. 2) resulted in an expansion of cTECs but a reduction in mTECs. One possible explanation for this finding could be that TEC precursors are preferentially differentiating into the cTEC lineage.

### T cell differentiation in *Eed*^fl/fl^::*β5tCre* mice is affected at early and late stages

**Fig. 3:**
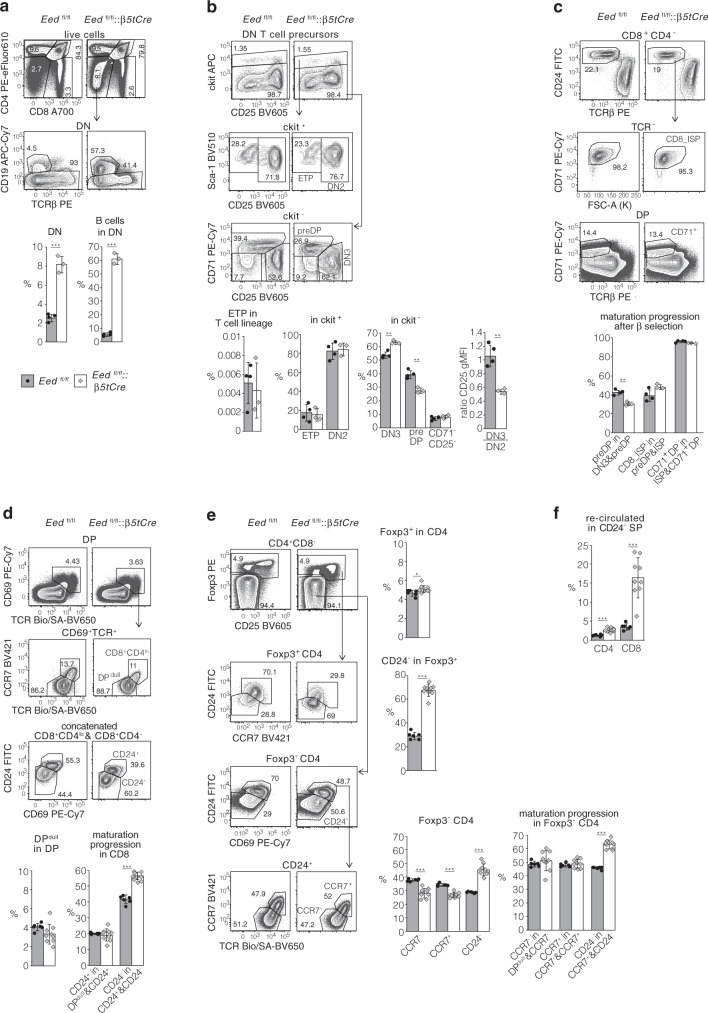
EED-deficient TEC affect thymocyte development at early and late stages. **a** Distribution of B cells and T lineage precursors within DN cells. **b** Quantification of ETP, DN2, DN3 and preDP subsets within DN T cell precursors. **c** Analysis of maturational progression from CD8 ISP to CD71^pos^ DP thymocyte stages. Characterization of the developmental progression within CD8 (**d**) and CD4 lineage (**e**) selected thymocytes. **f** Quantification of re-circulated peripheral T cells within mature CD24^neg^ SP subsets. Representative flow cytometry contour plots are shown, bar graphs display mean values with SD together with individual data points from *n* = 4 *Eed*^fl/fl^ and *n* = 3 *Eed*^fl/fl^:*:β5tCre* mice (**a**–**c**) or *n* = 6 *Eed*^fl/fl^ and *n* = 9 *Eed*^fl/fl^::*β5tCre* mice (**d**–**f**) at 3 weeks of age. ^*^p < .05, ^**^p < .01, ^***^p < .001 (two-tailed unpaired Student’s *t* test). Data in bar graphs are from one experiment representative of three independent experiments. *Eed*^fl/fl^::*β5tCre*: white bars with grey diamonds, *Eed*^fl/fl^ grey bars with black circles. *n* = biologically independent replicates per group. ^*^p < 0.05, ^**^p < 0.01, ^***^p < 0.001 (two-tailed unpaired Student’s *t* test). The gating for thymocyte subsets is displayed in Supplementary Fig. 3. Source data including exact statistical test values are provided in the Source Data file.

We next evaluated the thymopoiesis of 3-week-old *Eed*^fl/fl^ and *Eed*^fl/fl^::*β5tCre* mice (Supplementary Fig. 3a, b). In the absence of EED, the proportion of cells in the CD4^−^CD8^−^ double-negative (DN) thymoycte compartment increased 3–4-fold (2.5 ± 0.4% vs. 8.1 ± 0.9%; Fig. 3a). This relative increase was mainly due to the presence of B cells although their absolute numbers were not altered (4.8 ± 0.6 × 10^5^ in *Eed*^fl/fl^*::β5tCre* and 3.2 ± 1.3 × 10^5^ in *Eed*^fl/fl^). The relative number of early thymic progenitors (ETP, defined as DN CD44^hi^ckit^hi^Sca-1^pos^CD25^−^) and DN2 (DN CD44^hi^ckit^hi^Sca-1^pos^ CD25^+^) cells in relation to all thymocytes of the αβ T cell lineage (i.e. excluding cells positive for TCRγδ, CD19, CD11b, CD11c, DX5, NK1.1, Gr1, F4/80, Ter119 and MHCII) was comparable in mutant and wild-type mice (Fig. 3b and Supplementary Fig. 3a). We observed, however, a partial block at the DN3 (i.e. DN CD44^−^CD25^+^) β-selection checkpoint. Compared to DN2 cells, DN3 thymocytes exhibited an almost 2-fold reduction in CD25 expression, which was paralleled by a significant reduction in the frequency of CD71^+^ pre-double-positive (preDP) thymocytes in the ckit^neg^ DN subpopulation (27.5 ± 2.1% vs. 38.7 ± 2.8). The progression through subsequent maturation stages (i.e. CD71^+^CD8^+^ immature single-positive (ISP) and CD71^+^DP) was, however, not affected in mutant mice (Fig. 3c). Hence, these results indicate a partial block at the β-selection checkpoint caused by an EED deficiency in TEC. Whether the reduction in CD25 expression in DN3 is causally linked to the observed decrease in the generation of preDP thymocytes remains to be determined.

We next analysed positive selection into the single-positive (SP) thymocytes. Although the frequencies of positively selected DP^dull^ cells were unchanged, we detected in *Eed*:^fl/fl^:*β5tCre* mice an accumulation of both CD8SP and CD4SP (T_reg_ excluded) mature cells with a CD69^−^CD24^−^ phenotype (CD8SP: 56.5 ± 2.5% vs. 41.8 ± 2.6%; CD4SP: 45.4 ± 4.3% vs. 28.5 ± 0.5%, Fig. 3d, e). Both lineages displayed a normal progression from DP^dull^ to CD69^+^CD24^+^ immature SP stages, suggesting that the relative increase of mature CD24^−^ SP was caused by retention in the thymus and not by an altered developmental transition. Although the overall frequency of Foxp3^+^ T_reg_ cells within CD4 SP thymocytes was only marginally, yet significantly elevated in mutant mice, their phenotype was severely skewed towards a CD24^−^CCR7^lo^ expression pattern (67.4 ± 5.0% vs. 28.9 ± 3.2%) indicative of extended residence of cells in the medulla and/or re-circulation from the periphery back to the thymus (Fig. 3e). Within that CD24^−^ subset, the frequency of re-circulated peripheral T cells with a CD44^+^ phenotype (excluded from the above analysis) was significantly increased in *Eed*^fl/fl^::*β5tCre* mice, especially among CD8SP cells (CD24^−^CD8:^+^ 16.4 ± 5.3% vs. 3.5 ± 0.8%; CD24^−^CD4^+^: 2.7 ± 0.5% vs. 1.2 ± 0.2%, Fig. 3f and Supplementary Fig. 3b). Hence, the absence of PRC2 function in TEC caused an accumulation of specific SP thymocytes.

**Fig. 4:**
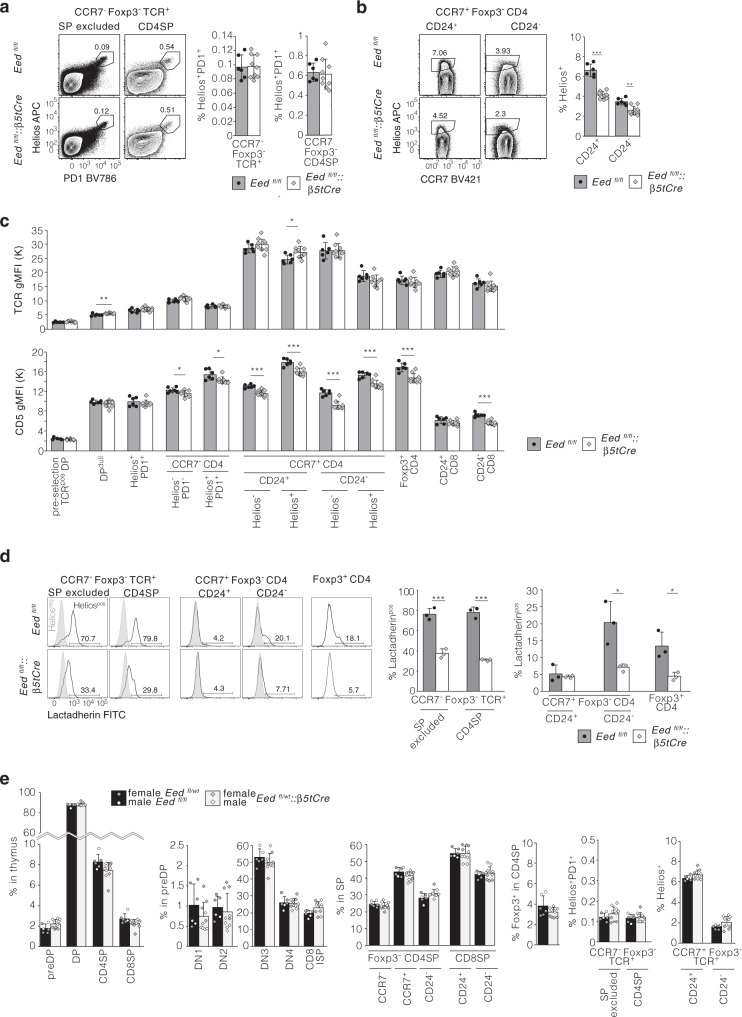
Negative selection and TCR avidity are reduced in a EED-deficient thymic microenvironment. Analysis of negative selection of CD4 lineage thymocytes in the cortex (**a**) and the medulla (**b**). CD5 and TCR geometric mean fluorescence intensity on indicated thymocyte subsets (**c**). Quantification of apoptotic cells in Helios-positive thymocyte subsets (**d**). Analysis of thymocyte development in *Eed*:^fl/wt^:*β5tCre* and control mice. Frequencies of preDP, DP and SP subsets, developmental stages within preDP thymocytes, maturation within SP cells, frequencies of Treg in CD4SP and frequencies of negatively selected cells (**e**). Representative flow cytometry contour and histogram plots are shown. Bar graphs display mean values with SD together with individual data points from *n* = 6 *Eed*^fl/fl^ and *n* = 9 *Eed*^fl/fl^::*β5tCre* mice at 3 weeks of age (**a**–**c**) or *n* = 3 *Eed*^fl/fl^ and *n* = 3 *Eed*^fl/fl^:*:β5tCre* mice at 4 weeks of age (**d**) representative of at least two independent experiments. *Eed*^fl/fl^::*β5tCre*: white bars with grey diamonds, *Eed*^fl/fl^ grey bars with black circles. Data in (**e**) are pooled from two independent experiments with female (*n* = 4 *Eed*^fl/wt^:*:β5tCre*: light grey bars with grey diamonds, *n* = 4 *Eed*^fl/wt^ black bars with grey circles) and male mice (*n* = 6 *Eed*^fl/wt^:*:β5tCre*: light grey bars with white diamonds, *n* = 2 *Eed*^fl/fl^ black bars with white circles) at 4–6 weeks of age. *n* = biologically independent replicates per group. ^*^p < 0.05, ^**^p < 0.01, ^***^p < 0.001 (two-tailed unpaired Student’s *t* test). The gating for thymocyte subsets is displayed in Supplementary Fig. 3. Source data including exact statistical test values are provided in the Source Data file.

Next, we characterized the negative selection of CD4 lineage thymocytes. The first wave of negative selection within the cortex (as identified by the co-expression of Helios and PD1 on Foxp3^−^ and CCR7^−^ TCR^+^ DP or CD4SP thymocytes) was undisturbed by the absence of EED in TEC (Fig. 4a). The second wave, which takes place in the medulla and is characterized by Helios expression on Foxp3^−^ CD4SP thymocytes^27^, was reduced (CD24^+^: 4.1 ± 0.4% vs. 6.6 ± 0.6%; CD24^−^: 2.5 ± 0.3% vs. 3.4 ± 0.4%, Fig. 4b). This reduction correlated with both lower CD5 expression on SP thymocytes (Fig. 4c) and significantly fewer apoptotic cells present in the Helios-positive subsets (Fig. 4d) suggestive of having received TCR-mediated signals of too low avidity to commit to programmed cell death^28^. In contrast, we observed no differences in thymocyte selection and maturation in *Eed*:^fl/wt^:*β5tCre* mice indicating that the presence of one *Eed* allele in TEC is sufficient to instruct regular T cell development (Fig. 4e).

Under physiological conditions, apoptotic cells are rapidly engulfed and cleared by thymic macrophages (Mø)^29^ and conventional Type 1 DCs (cDC1)^30^. Thymus immigrating DCs and B cells can also provide peripheral antigens for central tolerance induction^9–11^. We therefore determined the frequencies of thymic Mø, B cell and DC subsets in control and *Eed*^fl/fl^::*β5tCre* mice and found that their relative frequency was significantly increased in mutant animals (Supplementary Fig. 4). Increased Mø and cDC1 frequencies also indicated effective removal of apoptotic cells^11,30,31^ thus providing a cellular mechanism for the reduced detection of cells having received negative selection signals. In addition, the relative increase in B cells and thymus immigrating DCs could compensate for defects in selection mechanisms by PRC-deficient mTECs. Thus, the frequency of detectable negatively selected thymocytes was significantly reduced in the medulla, but not the cortex, of *Eed*^fl/fl^::*β5tCre* mice.

### The TCR repertoire in single-positive thymocytes of *Eed*^fl/fl^::β5tCre mice is narrowed

**Fig. 5:**
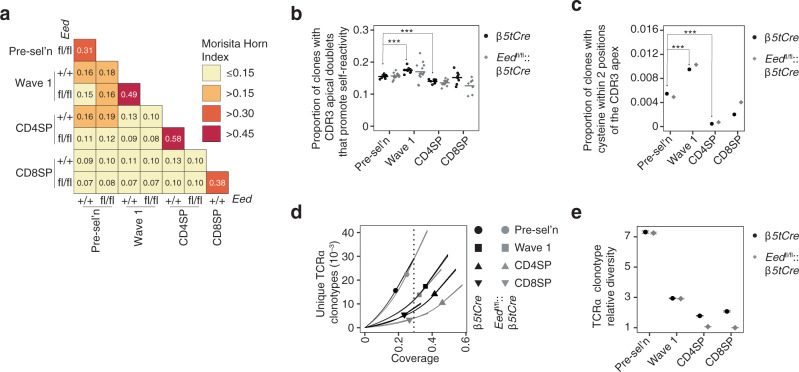
TCRα repertoire analysis of YAe62 TCRβ-transgenic thymocytes in *β5tCre* or *Eed*^fl/fl^*::β5tCre* bone marrow chimeras. Unless stated otherwise, the analyses compared TCR catalogues, which were formed by aggregating samples of a given T cell subset/genotype combination. The *β5tCre* and *Eed*^fl/fl^*::β5tCre* TCR catalogues contained the following numbers of samples: 9 and 14 mice, respectively (pre-selection, Wave 1 and CD8SP); 8 and 14 mice, respectively (CD4SP). **a** Heatmap shows the Morisita-Horn index for each pair of TCR catalogues. **b, c** Amino acid composition of the CDR3 apex. Considering the amino acid doublets at CDR3 positions 6 and 7, (**b**) shows the proportion of TCRα clones with doublets that promote self-reactivity^33^. Each symbol represents an individual sample. Samples with fewer than 100 clones were excluded, (**c**) depicts the proportion of TCRα clones with cysteine anywhere from CDR3 position −2 to +2 as determined by apex-out CDR3 alignment^35^. ^***^p = 4.3 × 10^−5^ (pre-selection vs. Wave 1) and *p* = 1.7 × 10 ^−6^ (pre-selection vs. CD4SP) using Student’s *t* test (unpaired, two-sided, Bonferroni-corrected) in (**b**) or *p* = 1.5 × 10^−6^ (pre-selection vs. Wave 1) and *p* = 2.8 × 10^−15^ using Fisher’s exact test (2 × 2 table, two-sided, Bonferroni-corrected) in (**c**). **d**, **e** TCRα clonotype diversities. **d** Cumulative number of unique TCRα clonotypes plotted as a function of “coverage”, defined as the proportion of all clones (encountered and unencountered) in a given T cell subset that belong to clonotypes encountered in the catalogue. Symbols show the observed values and curves outline the 95% confidence intervals of the rarefaction and extrapolation curves determined using 10 bootstrap replications. **e** Relative diversity of TCR catalogues. Symbols indicate ratios of the numbers of unique TCRα clonotypes per TCR catalogue at the level of coverage indicated by the vertical dashed line in (**d**). Error bars in (**e**) show the 95% confidence intervals of the relative diversities of TCR catalogues based on 10 bootstrap replications.

We extended the analyses of thymocyte selection by characterizing the TCR repertoire selected in the presence of EED-deficient TEC. *Eed*^fl/fl^::*β5tCre* and *β5tCre* (CD45.2) mice were transplanted with T cell-depleted bone marrow cells from mice transgenic for the YAe62 TCR β chain (TCRVb8.2)^32^. Donor-derived (CD45.1) thymocytes at distinct developmental stages (see Supplementary Fig. 5a) were isolated 4 weeks after transplantation and their TCRα chain repertoire was sequenced. Each T cell lineage showed a high degree of TCRα repertoire overlap between mutant and control samples, and the T cell lineages were equally distinct from each other regardless of the PRC2-deficiency (Fig. 5a and Supplementary Fig. 5). Certain amino acid doublets and cysteine promote T cell self-reactivity when present at the apex of the complementarity-determining region 3 (CDR3) of the TCRα or TCRβ chain^33–35^. The frequency of TCRα clonotypes bearing these amino acid motifs varied between T cell lineages^33–35^ but did not differ significantly between mutant and control samples (Fig. 5b, c). Except for a distal shift in *Traj* segment usage by DP thymocytes in mutant mice, *Trav* and *Traj* gene segment usage and CDR3 length distributions were comparable to controls (Supplementary Fig. 5). This shift in *Traj* may reflect a longer sojourn in the cortex due to the presence of a larger cTEC scaffold relative to the thymocyte pool in EED-mutant mice. Whereas the diversity of the pre-selection and wave 1 TCRα repertoires was normal, the CD4SP and CD8SP TCRα repertoires selected by EED-deficient TEC showed a 2-fold lower diversity than controls (Fig. 5d, e). These results indicate that EED deficiency did significantly narrow the TCRα repertoires of CD4SP and CD8SP thymocytes.

### Increased frequency of activated T_reg_ cells in *Eed*^fl/fl^::*β5tCre* mice

**Fig. 6:**
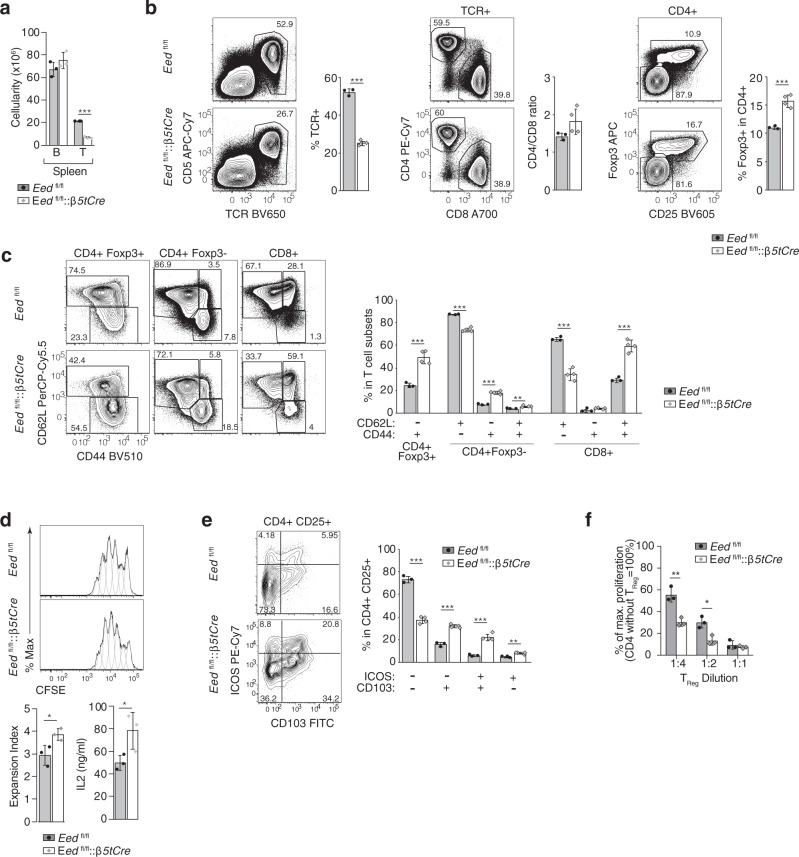
Peripheral T cells and their function in *Eed*^fl/fl^::*β5tCre* and *Eed*^fl/fl^ mice. **a** Absolute numbers of splenic B and T cells in *Eed*^fl/fl^ (*n* = 3) and *Eed*^fl/fl^*::β5tCre* mice (*n* = 3) at 4 weeks of age. **b** Representative contour plots and frequencies of lymph-node-derived T cell subsets. Frequency of TCRβ+ T cells, the CD4/CD8 ratio within TCRβ+ T cells and percentage of Foxp3^+^ T_Reg_ in CD4 T cells. **c** Frequencies of activated cells (CD44^+^CD62L^−^) in Foxp3^+^ T_Reg_ and frequencies of naive (CD44^−^CD62L^+^), central memory (CD44^+^CD62L^+^) and effector memory (CD44^+^CD62L^−^) T cells within the populations of Foxp3^−^ CD4 and CD8 lymph node cells. **b**, **c** *Eed*^fl/fl^ (*n* = 3) and *Eed:*^fl/fl^*:β5tCre* mice (*n* = 4) at 4 weeks of age. **d** Proliferative response and IL-2 secretion of naive lymph node CD4 T cells (CD44^lo^CD25^−^) from *Eed*^fl/fl^ (*n* = 3) and *Eed*^fl/fl^*::β5tCre* mice (*n* = 3) at 4 weeks of age. Representative histograms of CFSE dilution (including model components used for expansion index calculations; grey dotted lines) following stimulation for three days with CD3 mAb (1 µg/ml) and irradiated Rag2^−/−^ splenocytes. Bar graphs display the mean expansion index (left) and mean IL2 concentrations in the supernatant (right) with SD. **e** Representative contour plots and frequencies of lymph-node-derived T_Reg_ subsets defined by their differential expression of ICOS and CD103 from *Eed*^fl/fl^ (*n* = 3) and *Eed*^fl/fl^::*β5tCre* mice (*n* = 4) at 4 weeks of age. **f** T cell suppression assay: CD3 mAb-induced proliferation of sorted naive WT CD4 T cells (CD44^lo^CD25^−^) in the presence of sorted lymph node Treg (CD4^+^CD25^+^) from *Eed*^fl/fl^ (*n* = 3) and from pools of two *Eed*^fl/fl^::*β5tCre* mice each (*n* = 3) of mixed sex at 7–9 weeks of age. ^*^p < 0.05, ^**^p < 0.01, ^***^p < 0.001 (unpaired Student’s *t* test). Data in graphs show mean ± SD and are representative of two independent experiments. Contour plots (**b, c, e**) and histograms (**d**) are representative of data in bar graphs. *Eed*^fl/fl^::*β5tCre*: white bars with grey diamonds, *Eed*^fl/fl^ grey bars with black circles. *n* = biologically independent replicates per group. Source data including exact statistical test values are provided in the Source Data file.

The peripheral T cell compartment of *Eed*^fl/fl^::*β5tCre* mice demonstrated a T cell lymphopenia, equally affecting CD4 and CD8 T cells, (Fig. 6a, b). This resulted in a reduced frequency of naive and an expansion of central and effector memory T cells (Fig. 6c). Naive T cells from *Eed*^fl/fl^::*β5tCre* mice neither showed a deficiency in proliferation nor IL-2 production in response to CD3 mAb and co-stimulatory signals (Fig. 6d).

We next determined the frequency and activation state of T_reg_ cells. In comparison to controls, mutant mice showed a significant increase in the frequency of T_reg_ among CD4 cells (Fig. 6b) with a larger proportion of these cells displaying an activated phenotype, as revealed by a higher frequency of cells negative for CD62L (Fig. 6c) and positive for CD103 and/or ICOS (Fig. 6e). In comparison to control mice, peripheral T_reg_ cells from *Eed*^fl/fl^::*β5tCre* mice exerted a higher potency suppressing in vitro the proliferation of mitogenically activated CD4 effector cells (Fig. 6f). Taken together, these findings indicate that the increased frequency and higher suppressive capacity of T_reg_ cells in *Eed*^fl/fl^::*β5tCre* mice likely control a dysregulated immune response as a result of either by lymphopenia or self-reactivity.

### *Eed*^fl/fl^*::ZsGreen::β5tCre* mice contain mTEC with PRC2 activity

**Fig. 7:**
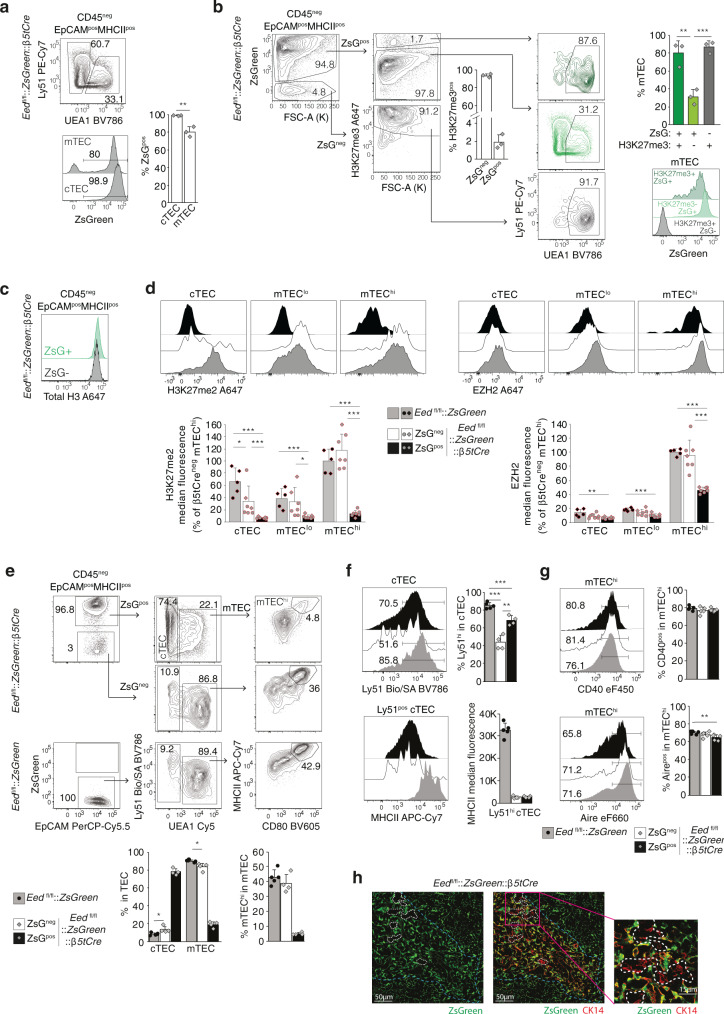
*Eed*^fl/fl^::*ZsGreen*::*β5tCre* mice contain a subpopulation of Eed-proficient mTEC. **a** ZsGreen expression in cTEC and mTEC isolated from 4-week-old *Eed*^fl/fl^:*:ZsGreen*::*β5tCre* mice. **b** Quantification of H3K27me3 abundance in ZsGreen-positive and -negative TEC and their phenotypic characterization. **c** Representative histograms of histone H3 abundance in ZsGreen-positive and -negative TEC. **d** Representative histograms and median fluorescence quantification of H3K27me2 and EZH2. Fluorescence of control mTEC^hi^ was set as 100%. Data are pooled from two independent experiments identified by circles and diamonds, respectively. **e** Representative FACS plots and quantitative analysis of cTEC, mTEC and mTEC^hi^ frequencies within ZsGreen-positive and -negative TEC. **f**, **g** Representative histograms and quantitative analysis of ZsGreen-positive and -negative cTEC (**f**) and mTEC^hi^ (**g**) phenotypes. **h** Spatial distribution of ZsGreen-positive and -negative TEC in thymus tissue sections of 4-week-old *Eed*^fl/fl^:*:ZsGreen*::*β5tCre* mice. Immunofluorescence analysis of mTEC expressing ZsGreen and cytokeratin 14 (CK14) in the medulla (marked by dashed blue line) identifying single cells and small aggregates of ZsGreen-negative cells (white dashed lines). Data in bar graphs show mean ± SD and are pooled from two independent experiments (**a**, **b**, **d**) or are representative of two independent experiments (**e**–**g**). Contour plots and histograms are representative for data displayed as frequencies in bar graphs (**a**, **b**, **d**, **e**–**g**). Immunofluorescence data are representative of two independent experiments with 3 sections each. **a**, **b** *Eed*^fl/fl^*::ZsGreen::β5tCre* (*n* = 3), in (**b**) dark green represents ZsGreen-positive H3K27me3-positive, light green shows ZsGreen-positive H3K27me3-negative, and grey depicts ZsGreen-negative H3K27me3-positive, (**d**) *Eed*^fl/fl^::*ZsGreen* (*n* = 5, grey histograms and bars with black circles and diamonds) *Eed*^fl/fl^*::ZsGreen::β5tCre* (*n* = 7, ZsGreen-negative: white histograms and bars with grey circles and diamonds, ZsGreen-positive: black histograms and bars with grey circles and diamonds), (**e**–**g**) *Eed*^fl/fl^:*:ZsGreen* (*n* = 5, grey histograms and bars with black circles), *Eed*^fl/fl^*::ZsGreen::β5tCre* (*n* = 5, ZsGreen-negative: white histograms and bars with grey diamonds, ZsGreen-positive: black histograms and bars with grey diamonds); *n* = biologically independent replicates per group, mice were 3–4 weeks of age. ^*^p < 0.05, ^**^p < 0.01, ^***^p < 0.001 (two-tailed unpaired Student’s *t* test). Source data including exact statistical test values are provided in the Source Data file.

TEC isolated from *Eed:*^fl/fl^*:β5tCre* mice contained, as noted above (Fig.1f), a subpopulation that had escaped gene deletion and thus maintained its H3K27me3 marks. These “EED escapees” were predominantly mTEC, a finding that was also observed in *Ezh1*^*ko*^::*Ezh2*^fl/fl^::*β5tCre* mice (Supplementary Fig. 2). This result was unexpected as Cre expression under the *Psmb11* transcriptional control targets >99.5 % of all mTECs^26^. To exclude a lack of Cre activity as an explanation for the detection of mTEC with regular H3K27me3 marks, we next generated *Eed*^fl/fl^::*ZsGreen*::*β5tCre* mice where ZsGreen expression depends on the Cre-mediated removal of a floxed stop-cassette 5ʹ of the transgenic reporter. In these mice, 98.6 ± 0.3% of all cTEC, yet only 80.3 ± 5.6% of all mTEC expressed ZsGreen (Fig. 7a). The loss of ZsGreen expression coincided with the detection of H3K27me3 marks in 93.2 ± 2% of all TEC, whereas the vast majority of ZsGreen^+^ TEC lacked H3K27me3 (97.4 ± 0.6%; Fig. 7b) although their total histone H3 abundance was comparable (Fig. 7c). Furthermore, H3K27me2 median fluorescence was drastically reduced in ZsGreen^+^ but not ZsGreen^−^ mTEC subsets (mTEClo: ZsG^+^ 8.5 ± 3.0%, ZsG^−^ 30.1 ± 23.5%, control 38.6 ± 16.0%; mTEChi: ZsG^+^ 13.7 ± 5.2%, ZsG^−^ 115.6 ± 28.6%, control 100 ± 19.6%; Fig. 7d, left panels). In addition, the lack of EED correlated with a 2-fold reduction in EZH2 fluorescence (cTEC: ZsG^+^ 6.8 ± 1.5%, control 14.2 ± 5.2%; mTEClo: ZsG^+^ 10.2 ± 2.9%, control 18.6 ± 2.0%; mTEChi: 46.1 ± 4.2%, control 100 ± 4.5%; Fig. 7d, right panels). However, a minor fraction of ZsGreen^+^ and H3K27me3^+^ TEC were detected (1.9 ± 0.8%; Fig. 7b) that were predominantly mTEC but displayed lower amounts of ZsGreen (Fig. 7b). This phenotype is compatible with promiscuous *β5tCre* expression in “EED mTEC escapees”. Hence, most H3K27me3^+^ “EED escapees” must be the progeny of rare TEC progenitors that do not express Cre.

*Eed*^fl/fl^::*ZsGreen*::*β5tCre* mice provided an opportunity to compare in the same thymus ZsGreen-positive EED-deficient and ZsGreen-negative EED-proficient TEC (aka “EED escapees”). ZsGreen-negative cTEC had a reduced Ly51 and MHC class II expression, thus providing a phenotype distinct from that of ZsGreen-positive cTEC (Fig. 7f). mTEC and the contingent of mature mTEChi demonstrated markedly reduced frequencies in the absence PRC2 activity in contrast to corresponding cells among “EED escapees” (Fig. 7e). The frequency of CD40^+^ EED-deficient mTEChi remained unchanged but that of AIRE^+^ cells was reduced when compared to controls (Fig. 7g). The maturational progression of EED-deficient mTEC thus progressively declined, whereas that of “EED escapees” was comparable to control mTEC. Both ZsGreen-positive and -negative mTEC were uniformly distributed throughout the medulla of *Eed*^fl/fl^::*ZsGreen*::*β5tCre* mice, yet the latter cell type occasionally formed small cell clusters (Fig. 7h). Taken together, these results are compatible with the notion that EED-proficient mTEC had most likely arisen via a non-canonical differentiation path unrelated to ineffective Cre recombination.

### EED-competent mTEC in *Eed*^fl/fl^::*β5tCre* mice represent a novel, non-canonical mTEC differentiation pathway

**Fig. 8:**
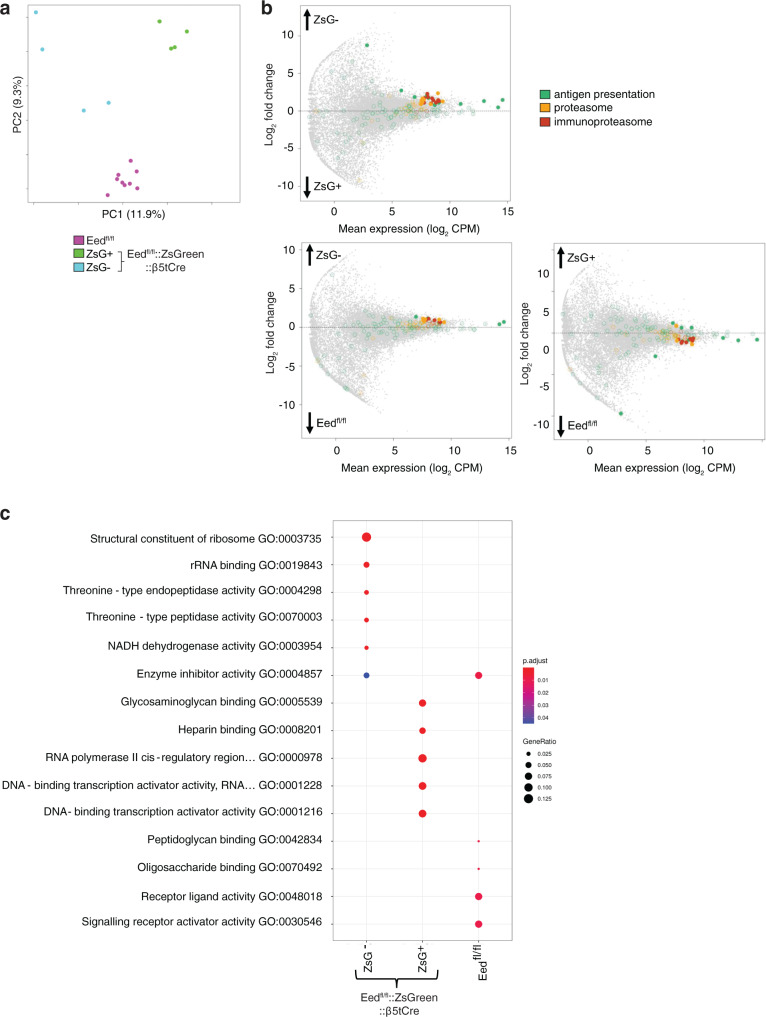
ZsGreen-positive and -negative mTEC from *Eed*^fl/fl^::*ZsGreen*::*β5tCre* mice are transcriptomically distinct from mTEC isolated from *Eed*^fl/fl^ mice. **a** Principal component analysis of bulk RNA-seq samples. **b** Scatter plots of differentially expressed genes for each type of mTEC showing genes belonging to gene ontology terms related to antigen presentation and processing (GO:0019882 - green), proteasome (KEGG:03050 - yellow) and immunoproteasome (CORUM:39 - orange). CPM = counts per million. Dashed lines indicate a fold change of 1 and arrows indicate the direction of higher expression. Filled points are significant at FDR < 0.05. **c** A dot plot showing significantly enriched biological pathways within genes significantly upregulated in each type of mTEC.

In wild-type mice, mTECs are derived from progenitors that express *Psmb11*^26^. To test the hypothesis that “EED escapees” constitute a separate lineage, we next compared the transcriptome of ZsGreen-positive EED-deficient and ZsGreen-negative EED-proficient mTEChi. A principal component analysis of the gene expression profiles identified three distinct clusters with “EED escapees” mTEC^hi^ grouping separately from EED-deficient and *Eed*^fl/fl^*::ZsGreen* mTEChi, suggesting that ZsGreen-negative EED-proficient cells either represent a distinct, non-canonical TEC lineage or a distinct differentiation pathway ending in a similar, but nonetheless transcriptomically distinct, medullary compartment (Fig. 8a). Both ZsGreen-positive and -negative mTECs showed marked transcriptomic differences to *Eed*^fl/fl^*::ZsGreen* mTECs (518 and 305 genes, respectively, significant at FDR < 0.05). There were also transcriptomic differences between ZsGreen-positive and -negative mTECs (664 genes, significant at FDR < 0.05; Supplementary Fig. 6 and Supplementary Data 1–3). For example, genes encoding proteins involved in the immunoproteasome were expressed at lower copy numbers in the absence of EED (ZsGreen-positive vs. *Eed*^fl/fl^::*ZsGreen*: 1.58-fold, *p* < 0.002; ZsGreen-positive vs. ZsGreen-negative: 2.66-fold, *p* < 0.002; Fig. 8b, c). Endopeptidase activity was particularly marked in the ZsGreen-negative mTECs, with the highest expression of 12 components of the proteasome seen in these cells. Conversely, ZsGreen-positive mTECs had elevated expression of genes within pathways regulating transcriptional activity related to cellular stress, such as *Fosb* and *Jun* (Fig. 8c). Moreover, genes that were differentially expressed between ZsGreen-positive and either ZsGreen-negative or *Eed*^fl/fl^ mTECs were also enriched within molecular pathways associated with antigen processing and presentation (Supplementary Fig. 7). These results suggested a deficiency in normal antigen processing in the absence of EED expression along with upregulation of pathways indicative of cellular stress.

### Differential gene expression in EED-deficient and EED-proficient mTEC of *Eed*^fl/fl^::*ZsGreen*::*β5tCre* mice

mTEC subpopulations provide an almost complete molecular representation of TRAs with some of them being expressed at low frequency and in low copy number^8^. This capacity is aided by the transcriptional facilitator AIRE, which promiscuously regulates the expression of genes marked at their transcription start site (TSS) by H3K27me3^8^. We therefore determined at single-cell resolution whether EED-deficient and -proficient mTECs from *Eed*^fl/fl^::*ZsGreen*::*β5tCre* mice maintained their normal transcriptional programmes. Single-cell analysis of cTECs showed a clear separation between EED-deficient and *Eed*^fl/fl^ cells based on changes in transcripts associated with antigen processing and presentation (including *Psmb11* and *Ctsl*; Supplementary Fig. 8). Significant differences were observed in the clustering centroids of each of the three separate mTEC^hi^ populations analysed (all *p* < 0.05), further supporting the notion that EED-proficient mTEC in *Eed*^fl/fl^::*ZsGreen*::*β5tCre* mice represent the emergence of a non-canonical mTEC lineage (Supplementary Fig. 9).

**Fig. 9:**
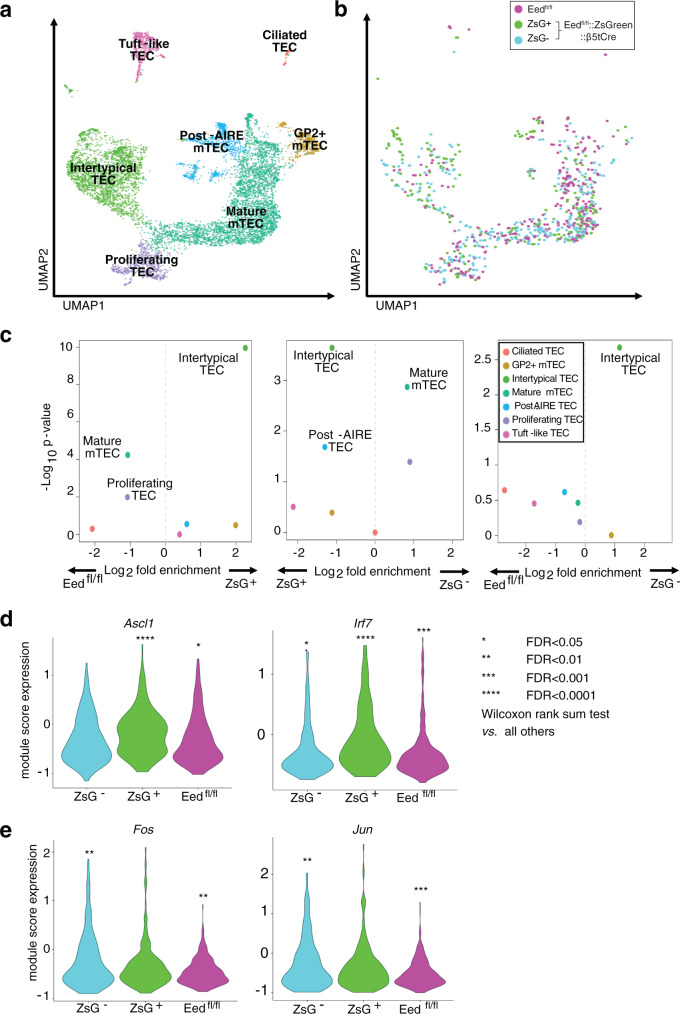
Single-cell annotation of mTEC lineages. **a** A UMAP plot showing all datasets projected together. mTEC cell type annotation was inferred from the combination of the two reference datasets. **b** The same UMAP projection showing only single-cell RNA-seq data on mTECs from this study. *Eed*^fl/fl^ (magenta), *Eed*^fl/fl^*::ZsGreen::β5tCre*: ZsGreen-positive (green) and ZsGreen-negative (blue). **c** Volcano plots showing enrichment of TEC subtypes for (left panel) *Eed*^fl/fl^*::ZsGreen::β5tCre* ZsGreen^+^ vs. *Eed*^fl/fl^ mTEC^hi^, (middle panel) *Eed:*^fl/fl^*:ZsGreen::β5tCre* ZsGreen^+^ vs. *Eed*^fl/fl^*::ZsGreen::β5tCre* ZsGreen^−^ mTEC^hi^ and (right panel) *Eed*^fl/fl^*::ZsGreen::β5tCre* ZsGreen^−^ vs. *Eed*^fl/fl^ mTEC^hi^. All TEC subtypes showing nominally significant differences are labelled. Violin plots showing transcription factor network expression for transcription factor networks upregulated in (**d**) *Eed:*^fl/fl^*:ZsGreen::β5tCre* ZsGreen^+^ mTEC^hi^, and (**e**) *Eed*^fl/fl^*::ZsGreen::β5tCre* ZsGreen^−^ mTEC^hi^. *Eed*^fl/fl^ (magenta), *Eed*^fl/fl^*::ZsGreen::β5tCre*: ZsGreen-positive (green) and ZsGreen-negative (blue). Source data including exact statistical test values are provided in the Source Data file.

Integration with existing single-cell mTEC transcriptomic datasets enabled us to further delineate mTEC differentiation in EED-deficient and -proficient mTEC^hi^ (Fig. 9a, b)^36,37^. EED-deficient mTEC^hi^ from *Eed*^fl/fl^::*ZsGreen*::*β5tCre* mice were diverted away from mature mTEC into an immature, intertypical TEC phenotype (Fig. 9c, left and middle panel). Conversely, EED-proficient mTEC^hi^ from *Eed*^fl/fl^::*ZsGreen*::*β5tCre* mice were able to differentiate into mature mTEC similarly to *Eed*^fl/fl^ mice but with a significant expansion of intertypical TEC (Fig. 9c, right panel). We used gene-regulatory network inference to identify transcription factor networks driving differences in mTEC differentiation and found *Ascl1* and *Irf7* network expression was significantly increased in EED-deficient mTEC^hi^ (Fig. 9d). *Jun* and *Fos* network expression was specifically elevated in EED-proficient mTEC^hi^ from *Eed*^fl/fl^::*ZsGreen*::*β5tCre* mice relative to *Eed*^fl/fl^ mice (Fig. 9e). This confirmed that EED-deficiency diverted mTEC differentiation towards a more immature medullary phenotype, whereas the majority of “EED escapees”, despite not originating from the canonical β5t^+^ TEC progenitor pool, were able to adopt a mature mTEC identity, although with several differences in transcription factor network expression.

**Fig. 10:**
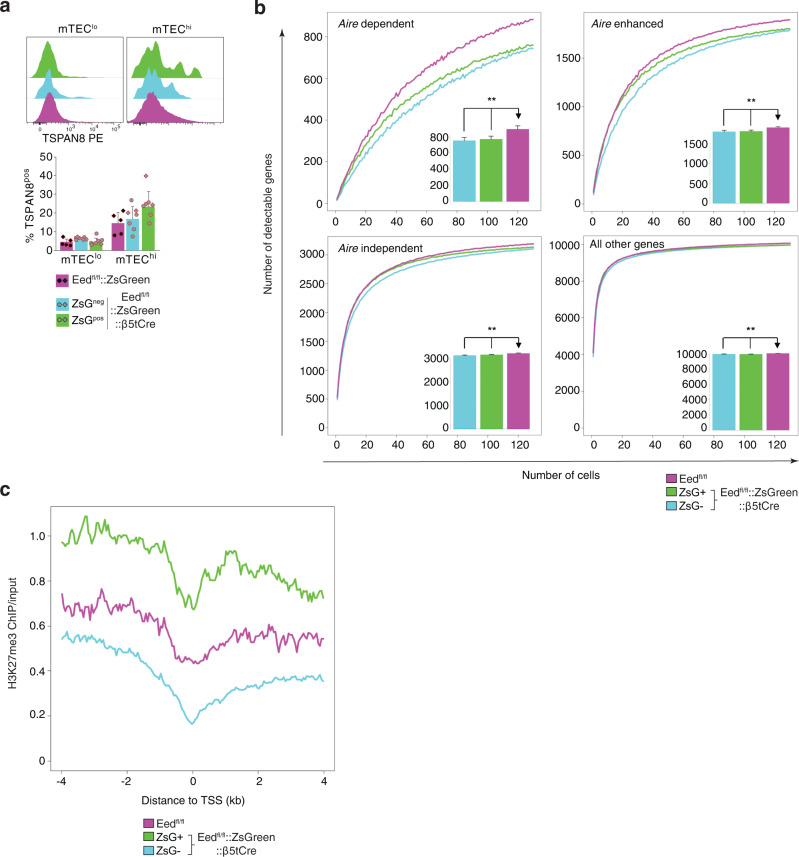
The effects of *Eed* loss on AIRE-dependent gene expression and H3K27me3 repressed genes. **a** Representative histograms and quantitative analysis of TSPAN8 expression in mTEClo and mTEChi. Graph shows mean with SD, data are pooled from two independent experiments identified by circles and diamonds, respectively. *Eed*^fl/fl^::*ZsGreen* (*n* = 5, magenta) *Eed*^fl/fl^*::ZsGreen::β5tCre* (*n* = 7 ZsGreen-negative: blue, ZsGreen-positive: green). *n* = biologically independent replicates per group, mice were 3–4 weeks of age. **b** The number of detectable genes from randomly sampled single ZsGreen-negative (blue), ZsGreen-positive (green) and *Eed*^fl/fl^ (purple) mTEC^hi^. The inset bar graphs show the mean number of detectable genes when resampling 130 single mTEC^hi^. Error bars indicate the standard deviation. ^**^Corrected *p* < 0.001 (two-sided Wilcoxon rank sum test with Benjamini-Hochberg correction). Prior to analysis, cells were downsampled to 200,000 transcriptomic fragments. **c** Median wild-type H3K27me3 ChIP/input in 50 bp bins around the transcription start site (TSS) for genes significantly upregulated in mTEC^hi^. Genes were those most highly expressed in ZsGreen-positive (green), ZsGreen-negative (blue) or *Eed*^fl/fl^ (magenta). Comparisons between ZsGreen-positive vs. ZsGreen-negative and ZsGreen-positive vs. *Eed*^fl/fl^ mTEC^hi^ revealed significant differences (*p* < 0.01). Source data including exact statistical test values are provided in the Source Data file.

We next assessed the consequences of the loss of EED on promiscuous expression of genes encoding TRAs whose transcripts depend on AIRE expression, are enhanced by AIRE or are generated independently of AIRE expression^8^. There were small changes in the overall expression of AIRE-controlled genes between EED-deficient and -proficient mTEC (Supplementary Fig. 10a). For example, AIRE-dependent TSPAN8 expression remained unchanged since this TRA was detected at comparable level in ZsGreen-positive and -negative mTEChi of experimental mice and their controls (Fig. 10a). By randomly sampling *AIRE* expressing single cells, we estimated for each of the different mTEC populations the number of detectable genes encoding TRAs (Fig. 10b). The breadth of promiscuous TRA expression was reduced in EED-deficient mTECs, affecting particularly AIRE-controlled loci (AIRE-dependent: 0.86-fold, *p* < 0.0001; AIRE-enhanced: 0.96-fold, *p* < 0.0001; AIRE-independent TRA: 0.98-fold, *p* < 0.0001; all other genes: 0.99-fold, *p* < 0.0001). A similar change in the pattern of TRA expression was observed in ZsGreen-negative mTEC isolated from *Eed*^fl/fl^::*ZsGreen*::*β5tCre* mice (AIRE-dependent: 0.84-fold, *p* < 0.0001; AIRE-enhanced: 0.94-fold, *p* < 0.0001; AIRE-independent TRA: 0.97-fold, *p* < 0.0001; all other genes: 0.99-fold, *p* < 0.0001). Hence, there was a subtle reduction in the breadth of promiscuous gene expression in mTEChi proficient or deficient in PRC2 activity within the same thymic environment. However, multivariate analysis showed that the presence of H3K27me3 marks near the TSS predicted whether or not a gene was upregulated in EED-deficient mTECs relative to those that were EED-proficient independently of whether or not that gene was regulated by AIRE (Supplementary Fig. 10b). This finding suggests that at least a portion of the mild deficiency in promiscuous gene expression was more likely to be driven by thymic microenvironmental changes than a direct effect of EED-deficiency. *Eed*^fl/fl^::*β5tCre* mice did not develop auto-antibodies or overt organ infiltration nor did they reveal signs of autoimmunity after antibody-mediated T cell depletion and subsequent endogenous T cell reconstitution (Supplementary Fig. 11).

Given the near-complete loss of H3K27me3 marks in ZsGreen-positive EED-deficient mTEC, we assessed the distribution of H3K27me3 ChIP-seq signal in wild-type mTEC^hi^ around those genes that were differentially expressed between separate mTEC populations. H3K27me3 ChIP-seq signals were significantly increased around the TSS of genes that were most highly expressed in ZsGreen-positive mTECs than in either of the two other TEC populations (H3K27me3 ChIP/input signal within 4 kb of the TSS: ZsGreen-positive vs. ZsGreen-negative: median 2.36-fold, FDR < 0.0001; ZsGreen-positive vs. *Eed*^fl/fl^ median 2.14-fold, FDR < 0.0001; Fig. 10c). Differences in the corresponding H3K27me3 ChIP-seq signals were, however, not observed between ZsGreen-negative and *Eed*^fl/fl^ mTEC (FDR > 0.05). Taken together, these data demonstrated that the loss of EED early in TEC development resulted in an mTEC compartment with a relatively modestly reduced capacity for promiscuous gene expression. This finding suggests that the expression of TRAs is not critically dependent on repressive chromatin modifications.

## Discussion

TEC development and function are dependent on EED, an essential component for assembly and activity of the PRC2 complex. The loss of EED in β5t-expressing TEC precursors and their progeny resulted in an absence of PRC2 activity in practically all cTEC (with the notable exception of few non-recombined cTEC displaying an atypical phenotype) and the majority of mTEC. However, up to 15% of all mTEC present in *Eed*^fl/fl^::*β5tCre* mice arise from an alternative mTEC developmental pathway originating from cells other than bi-potent, β5t-positive precursors and characterized by a distinct transcriptome, representing either a distinct TEC lineage or an alternate differentiation pathway into canonical mTEC cell types within an abnormal thymic microenvironment^26,38^. EED-proficient mTEC exhibits in *Eed*^fl/fl^::*β5tCre* mice a competitive growth/differentiation advantage over EED-deficient mTEC.

Despite the severely reduced cellularity of ETP and a partial block in β-selection, cTECs of *Eed*^fl/fl^*::β5tCre* mice support the differentiation to DP thymocytes and their positive selection despite differences in the expression of many genes required for antigen processing and presentation. Any deficiencies in antigen presentation may likely be compensated by the decreased thymocytes-to-cTEC ratio, thus lessening the competition between thymocytes and the normally limiting numbers of cTEC. This will result in an increased cross-talk between these two cell types and a longer sojourn of thymocytes in the cortex^39^. Indeed, the subtle differences in the choice of *Traj* segments within the TCRα repertoire of *Eed*^fl/fl^::*β5tCre* mice support this explanation as reflected by a more frequent use of distally positioned *Traj* family members^40^. Furthermore, the transition of thymocytes across the corticomedullary zone may be secondary to the decrease in *Psmb11* expression, as observed in β5t-deficient mice^41^.

The exact timing of the loss of EED expression in TEC determines thymus organogenesis. In a recently described mouse strain (*Eed*^fl/fl^::*Foxn1-Cre)*, an identical ablation of the *Eed* locus targeted to a time point early during TEC fate specification but prior to *Psmb11* expression hinders TEC maturation resulting in a severely dysplastic thymus in which PRC2’s role in development and function cannot be interrogated in detail^42^. Thus, it remains unknown whether mTEC development bypassing β5t-positive bi-potent TEC precursors can also occur in *Eed*^fl/fl^::*Foxn1-Cre* mice. We therefore conclude that the loss of EED expression early during TEC differentiation (i.e. before the emergence of bi-potent β5t-positive TEC precursors) and the consequent loss of canonical gene silencing by PRC2 precludes not only the normal progression along the canonical TEC differentiation pathway but also excludes the emergence of the alternative mTEC pathway described here.

In addition to intrathymic B cells, *Eed*^fl/fl^::*β5tCre* mice also show increased frequencies of thymic macrophages and DCs, although it is possible that this could simply be due to the observed reduction of thymocytes. The impaired antigen presentation by TEC may be compensated by immigrating DCs and B cells thus contributing to establishing central T cell tolerance^9–11^. Antigen transfer from mTEC to DCs may furthermore offset defects in mTEC-mediated antigen presentation^43^ and selecting a TCR repertoire with sufficient self-tolerance.

Signs of autoimmunity are not detected in *Eed*^fl/fl^::*β5tCre* mice despite an impaired expression of antigen processing pathways, a limited reduction in the representation of TRA in mTEC, a narrowed breadth of the TCR repertoire and peripheral T cell lymophopenia. Although reliant on mTEC and DCs for their selection^44–46^, T_reg_ generation is not offset by the reduced mTEC cellularity, indicating a likely compensation by the increased frequency of intrathymic DCs. The heightened frequency and function of peripheral T_reg_—resulting from lymphopenia-induced proliferation—contribute further to the observed lack of autoimmunity. Because peripheral T_reg_ cannot compensate for AIRE deficiency, the absence of autoimmunity corroborated our finding that EED deficiency results only in a minor alteration of TRA representation^47^.

Gene expression profiling of EED-deficient mTEChi demonstrated a significant upregulation of genes that are normally associated with H3K27me marks in EED-proficient mTEChi, both wild-type and “EED escapees”, a finding consistent with the removal of canonical gene silencing by PRC2. Changes in TEC development and composition are thus explained by gene de-repression in pathways involved in epithelial differentiation as highlighted in other organs, such as lung, including *Foxp1* and *Nfib* (Supplementary Fig. 7). This mechanism of de-repression was previously suspected as known PRC2 targets are characterized by active enhancer marks in immature but not mature mTEC^48^. The current study demonstrates that these chromatin changes are not merely correlative but that loss of PRC2 activity compromises mTEC progression to a mature phenotype, through the differential induction of transcription factor networks controlled by *Irf7* and *Ascl1*. Previous research had suggested that AIRE-regulated loci were marked by repressive chromatin modifications^20,49^. However, the relatively mild reduction in the breadth of TRA expression in *Eed*^fl/fl^::*β5tCre* mTECs suggests that H3K27me3 is not absolutely required for AIRE-regulated transcription. Rather, the role of PRC2 activity in TEC differentiation and suppression of alternative differentiation pathways raises the prospect that therapeutic manipulation of the TEC epigenome may be able to modulate negative selection in autoimmune diseases^50^.

## Methods

### Mice

C57BL/6 mice were obtained from Janvier. The generation of β5t-Cre, Eed, Ezh1 and Ezh2 conditionally targeted animals and TcRβ transgenic mice (YAe62β) have previously been reported^25,26,32,51^. Rosa26 knock-in mice that were engineered to contain the CAG-loxP-stop-loxP-ZsGreen sequence have been described elsewhere^52^. Experiments used animals with both sexes until 4 weeks of age, the gender is indicated for experiments with mice older than 4 weeks. Neonatal female Ly5.2 recipients of haematopoietic stem cells were conditioned with 200 cGy prior to receiving T cell-depleted Ly5.1 bone marrow cells (3 × 10^6^) from TCRβ transgenic mice (YAe62)^32^. Chimeric mice were analysed 4 weeks after engraftment. Rag2^−/−^ mice were bred and maintained in our institute’s SPF facility. Experimental (Cre-positive) and control (Cre-negative) mice were co-housed, C57BL/6 and β5t-Cre controls were bred separately. Mice were cared for and experiments were carried out in accordance with permissions and regulations of the Cantonal Veterinary Office of Basel-Stadt (licence 2737). All animals were kept under specific pathogen-free conditions at 22 ± 2 °C, 60 ± 15% humidity, and a 12 h light cycle with light phase beginning at 6 a.m. with a 30 min sunrise and ending at 6 p.m. with a 30 min sunset, with food and water ad libitum. Mice were euthanized by CO_2_ inhalation and decapitation.

### Thymic epithelial cell isolation

Thymic lobes were cleaned off the fatty tissues and incubated with Liberase and DNaseI (Roche Diagnostics; 200 and 30 μg/ml, respectively; 45 min, 37 °C) to obtain a cell suspension, which was subsequently filtered through a nylon mesh (100 µm pore size, Sefar Nitex) to remove debris. With the exception of Fig. 1e, wild-type TEC were magnetically enriched using the AutoMACS Pro Separator (Miltenyi Biotec) to obtain sufficient cells for analysis.

### Thymic macrophage and dendritic cell isolation

Cleaned thymic lobes were incubated with Collagenase D and DNaseI (Roche Diagnostics; 1 and 30 μg/ml, respectively; 45 min, 37 °C) to obtain a cell suspension, which was subsequently filtered through a nylon mesh (100 μm pore size, Sefar Nitex) to remove debris and stained.

### In vivo T cell depletion

In vivo T cell depletion was achieved by i.v. injection of a cocktail of CD4 (clone GK1.5; 200 µg), CD8 (53–67; 100 µg) and anti-Thy1.1 (T24; 50 µg) mAb.

### Flow cytometry

Thymocytes and TEC were stained as indicated, using antibodies and reagents listed in Supplementary Table 1, including dilutions. For intracellular staining, cells were fixed, permeabilized (Cytofix/Cytoperm Kit, BD Biosciences) and labelled except for Foxp3 staining, where the eBioscience transcription factor staining buffer set (ThermoFisher Scientific) was used. Stained samples were acquired on a FACSAria II or BD LSRFortessa flow cytometer with FACSDiva (BD Biosciences v8.01) and the data were analysed using the FlowJo (Treestar Inc. v9.8.3 and v10.7.1) software.

### In vivo cell proliferation assay

TEC proliferation was quantified by BrdU incorporation. In short, mice were i.p. injected with 1 mg BrdU (BD Pharmingen) diluted in 250 μl sterile PBS and analysed 16 h later by flow cytometry following the BrdU Flow Kit protocol (BD Biosciences).

### Histology

Frozen sections (8 µm) of OCT embedded thymus tissue were fixed in acetone and stained either with haematoxylin and eosin or with Abs as indicated (see Supplementary Table 1). For the detection of ZsGreen, samples were first fixed in 4% paraformaldehyde prior to embedding. The tissue was sectioned using the cryostat and attached to super-frost glass slides (ThermoFisher Scientific). Sections were then stained with primary antibodies (2 h, room temperature), washed and developed using secondary antibodies (1 h, room temperature). Images were acquired using a Zeiss LSM510 (Carl Zeiss).

### In vitro T cell proliferation assay

T cell subpopulations were labelled for 10 min at room temperature with CFSE (2.5 nM) and subsequently co-cultured for 72 h with irradiated (3000 cGy) Rag2^−/−^ splenocytes and 1 µg/ml of CD3 mAb. The proliferative response was evaluated by flow cytometry and assessed by the serial dilution of CFSE label.

### T cell suppression assay

Sorted naive WT lymph node CD44lo CD25neg CD4 T cells (50,000/well) were cultured together with irradiated (3000 cGy) Rag2^−/−^ splenic accessory cells (100,000/well) and the indicated amount of sorted lymph node CD4pos CD25pos T_reg_ in the presence of soluble CD3 mAb (0.5 μg/ml). After 48 h, 1 μCi ^3^H thymidine was added overnight and cells were harvested the following day.

### ELISA assay

The ELISA-Ready-Set-Go kit was used according to manufacturer’s protocol to determine the concentration of IL-2. The absorbance values of each well were then acquired using an absorbance reader (Biochrom) at 450 nm.

### RNA-seq and ChIP-seq

TECs were enriched as detailed above and then sorted using a FACSAria II (BD Bioscience). RNA was isolated using RNeasy kit (Qiagen) and subjected to sequencing (TrueSeq, BGI). Sequencing data are available through the Gene Expression Omnibus (GSE112050).

Adapter sequences were trimmed using Trimmomatic^53^. Reads were aligned against the Ensembl transcriptome and genome (GRCm38 plus ERCC spike-ins) using HISAT (version 0.1.6)^54^. Reads were allocated to protein-coding gene meta-features using HTSeq (intersection non-empty)^55^. Differential expression analysis on genes with mean expression at least 1 FPKM was conducted using general linear modelling in edgeR, using TMM normalization^56^. Genes were identified as differentially expressed using the default edgeR threshold (FDR < 0.05). Clustering was performed using principal component analysis. Gene ontology enrichment was conducted in gProfiler with enrichment values estimated from 1000 permutations^57^.

Single-cell sequencing data were pre-processed and aligned in the same manner. Low quality cells were identified by robust PCA outlier identification on several quality control metrics (alignment proportion, ERCC spike-in proportion, number of detectable genes, proportion of reads mapping to protein-coding genes, proportion of mitochondrial transcripts, proportion of ribosomal transcripts, 3ʹ-to-5ʹ coverage bias, transcriptomic variance, cell-to-mean correlation, the proportion of the library accounted for by the top 500 transcripts and GC content)^58^. Counts were adjusted between plates using ERCC spike-ins and then by library size using SCnorm^59^. Cells were clustered using the tSNE component of the linnorm package, specifying pre-normalized data^60^. scDD was used for differential analysis of single-cell RNA-seq data^61^. Richness of TRA expression was assessed by resampling the increasing number of single mTECs and calculating the mean number of genes expressed in different categories of genes. Each number of cells was resampled 100 times. Statistical significance was estimated using Wilcoxon rank sum tests and correcting for multiple hypothesis testing using the Benjamini-Hochberg method. Comparison to reference single-cell RNA-seq datasets was undertaken in Seurat, integrating the reference datasets together and then integrating the single-cell RNA-seq data from this manuscript onto that^36,37,62^. GENIE3 and RcisTarget were used to generate transcription factor gene-regulatory networks^63^. Module expression for genesets was calculated using Seurat.

ChIP-seq data were processed as previously described^64^. Briefly reads were pre-aligned using BWA (version 0.5.9) then high-quality alignments were processed using Stampy. Only reads with a MAPQ score of at least 10 were taken forward for further analysis. H3K27me3 peaks were called using irreproducibility discovery rate analysis (specifying broad peaks). Significance of ChIP/input changes between samples were estimated using Wilcoxon rank sum tests and correcting for multiple hypothesis testing using the Benjamini-Hochberg method.

RNA-seq and ChIP-seq data are available at the Gene Expression Omnibus (GSE112050 and GSE114713). AIRE ChIP-seq data were downloaded from GSE92597^65^.

### T cell sorting for TCR repertoire analysis

Whole-thymus suspensions were prepared by pushing organs through a 70 µm sieve into PBS containing 2% v/v heat-inactivated fetal calf serum and 2 mM EDTA (designated sort buffer). For CCR7 staining of thymocytes, suspensions were incubated for 30 min at 37 °C in pre-warmed sort buffer containing anti-CCR7. Cells were then pelleted by centrifugation and incubated for 30 min in sort buffer at 4 °C containing assortments of fluorochrome-conjugated monoclonal antibodies. After washing, cells were passed through a 40 µm sieve before sorting into 1.5 ml Eppendorf tubes containing 350 µl Qiagen Buffer RLT, which were frozen by pushing into dry ice and then stored at −80 °C until RNA isolation. Cell source, type and number are displayed in Supplementary Table 2.

### TCR sequence acquisition in YAe62-tg T cells

RNA was isolated using Qiagen RNeasy Mini kits with an elution volume of 22 µl, from which 12 µl were used to synthesize cDNA (Quantitect RT, QIAgen). Using 5 µl of cDNA template per reaction, TCRα transcripts were PCR amplified using a mix of 24 *Trav*-specific forward primers and a single *Trac*-specific reverse primer (Supplementary Table 3). Forward and reverse primers had distinct 5ʹ overhang adapter sequences that enabled addition of sample-specific indices and P5/P7 sequencing adapters in a second PCR using the Illumina Nextera XT DNA library preparation kit. Before the second PCR, magnetic beads (Agencourt AMPure XP, Beckman Coulter) were used to enrich amplicons >100 bp. Conditions for the first PCR were 98 °C for 5 min, 20 cycles of 98 °C for 10 s, 60 °C for 30 s and 72 °C for 30 s, followed by 72 °C for 2 min and for the second PCR were 72 °C for 3 min and 98 °C for 30 s, 20 cycles of 98 °C for 10 s, 63 °C for 30 s and 72 °C for 30 s, followed by 72 °C for 1 min. After determining amplicon concentrations using a QIAxcel capillary electrophoresis machine (Qiagen), equimolar amounts of amplicons from 46 samples were pooled into a single tube, concentrated using magnetic beads (Agencourt AMPure XP, Beckman Coulter) and then 300–500 bp amplicons were gel-purified before 250 bp paired-end sequencing on an Illumina MiSeq machine. The full TCR sequence dataset is available at the Sequence Read Archive (BioProject ID PRJNA565651) and processed data are available from the authors on request.

### TCR sequence processing

Sequences were aligned to *Trav* and *Traj* gene segments, and CDR3 amino acid sequences were ascertained, using molecular identifier groups-based error correction (MIGEC) software^66^. Subsequent analyses were performed using RStudio software. All data were filtered to exclude non-functional CDR3 sequences that were either out-of-frame or contained a stop codon. To avoid overestimating TCR diversity due to PCR or sequencing errors, sequences detected fewer than 3 times in any given sample were excluded from further analysis. Sequences that aligned with >1 *Trav* paralog were assumed to use the *Trav* paralog listed first by MIGEC except for Supplementary Fig. 5d, e, from which these ambiguous sequences were excluded. A unique combination of *Trav* segment and CDR3 amino acid sequence was defined as a clonotype. Each clonotype was counted only once per sample. All clonotypes detected in samples of a given combination of genotype and T cell subset were pooled to form a TCR catalogue. Unless otherwise indicated, analyses were performed at the TCR catalogue level. Self-reactive CDR3 doublet expression at positions 6 and 7 of the CDR3 was determined by calculating the proportion of sequences using any of the 175 amino acid doublets identified as promoting self-reactivity when present at this position. For this analysis only, position 1 of the CDR3 was designated as the conserved cysteine at the CDR3 N-terminus. For diversity analyses, the iNEXT software package was used to plot coverage-based rarefaction and extrapolation curves, plus relative diversity measurements, following the approach of Chao and Jost^67^ whose term “individual” we equate with “clone”, and “species” we equate with “clonotype”. Catalogues were assembled in a clone-based (individual-based) abundance format and clonotype (species) diversity estimates were made using the diversity order (Hill number or *q*). Morisita Horn indices were computed using EstimateS (Version 9.1.0, R. K. Colwell, http://purl.oclc.org/estimates) with clonotype abundance equal to the number of mice in which the clonotype was detected in a given TCR catalogue.

### TCRα chain analysis in non-transgenic T cells

TCRα repertoire analyses were performed by SMARTer 5ʹ RACE cDNA amplification on thymocytes as previously described^68^. CDR3 sequences were identified using LymAnalyzer^69^. Each unique TRAV–TRAJ clonotype was recorded in a FlowJo-based file to calculate frequencies of TRAV and TRAJ proximal and distal gene usage. Principal component analysis and hierarchical clustering (HC) were performed using FactomineR (http://factominer.free.fr/) and Factoextra (http://www.sthda.com/english/rpkgs/factoextra). The following 8 parameters were extracted from TCRα repertoire analysis for HC determination: relative frequencies of distal and proximal TRAV segments and frequencies of distal, medium, and distal TRAJ elements within proximal and distal TRAV containing transcripts.

### Statistical analyses

Statistical analyses for data presented were performed using Student’s *t* test (unpaired, two-tailed). *p* < 0.05 was considered significant. The statistical evaluation of RNA-seq data is described in separate statistical analysis section above. The sample size used and estimates of variation within groups were based on published results using similar approaches. No randomization was done for animal studies and investigators were not blinded to experimental group allocations.

### Reporting summary

Further information on research design is available in the Nature Research Reporting Summary linked to this article.

## Supplementary information

Supplementary information.

Reporting summary.

Description of Additional Supplementary Files.

Supplementary Data 1.

Supplementary Data 2.

Supplementary Data 3.

## Data Availability

The full TCR sequence dataset is available at the Sequence Read Archive (BioProject ID PRJNA565651) and processed data are available from the authors on request. RNA-seq and ChIP-seq data are available at the Gene Expression Omnibus (GSE112050 and GSE114713). AIRE ChIP-seq data were downloaded from GSE92597. Other data that support the findings of this study are available from the corresponding author upon reasonable request. Source data are provided with this paper.

## Source data

Source Data
