# Supplementary material for: Indispensable epigenetic control of thymic epithelial cell development and function by polycomb repressive complex 2
Source: Nat Commun. 2021 Jun 24;12:3933. doi: 10.1038/s41467-021-24158-w (PMC8225857; doi:10.1038/s41467-021-24158-w)
Supplement: Supplementary file 1 — Supplementary information. [file 41467_2021_24158_MOESM1_ESM.pdf]

Supplementary Information

Indispensable epigenetic control of thymic epithelial cell development and function by Polycomb Repressive Complex 2

Thomas Barthlott, Adam E. Handel, Hong Ying Teh, …, and Georg A. Holländer

Supplementary Figure 1

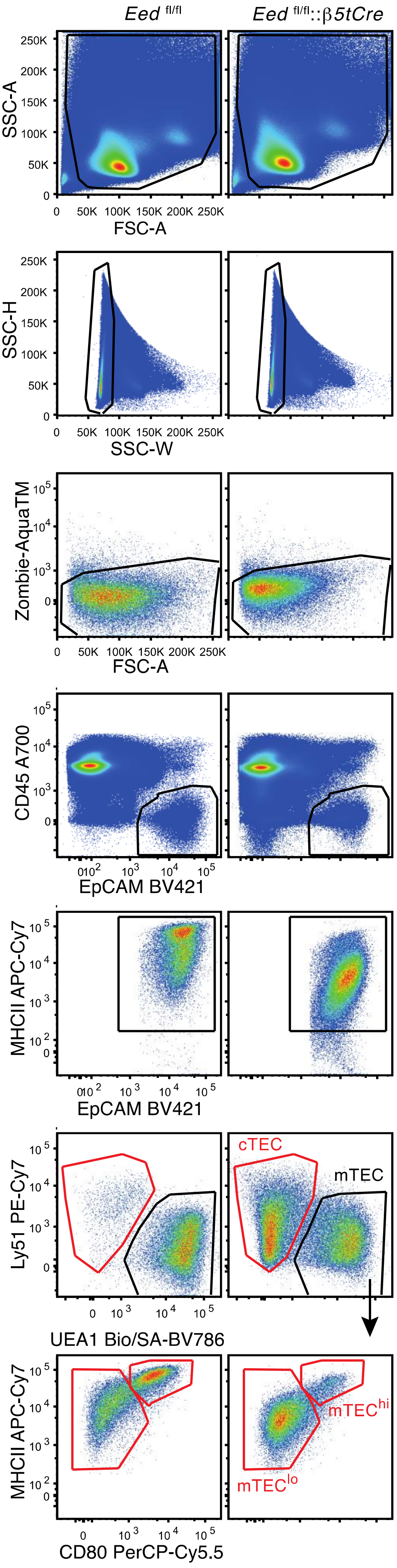

Supplementary Figure 1

TEC gating strategy. Events were first gated to eliminate cellular debris by forward scatter (FSC-A) and side scatter (SSC-A) which was followed by gating for single cells using the side scatter signal profile (height, SSC-H and width, SSC-W of signal). Live cells were then gated as events negative for Zombie Aqua^{TM}. The individual TEC were subsequently identified as negative for CD45 but positive for EpCAM and MHCII expression and phenotyped as cTEC (Ly51$^+$UEA1$^-$) and mTEC (UEA1$^+$Ly51$^-$). mTEC^{lo} and mTEC^{hi} were discriminated by MHCII and CD80 expression. Gates used for calculations and for display in Figure 2d are drawn in red.

Supplementary Figure 2

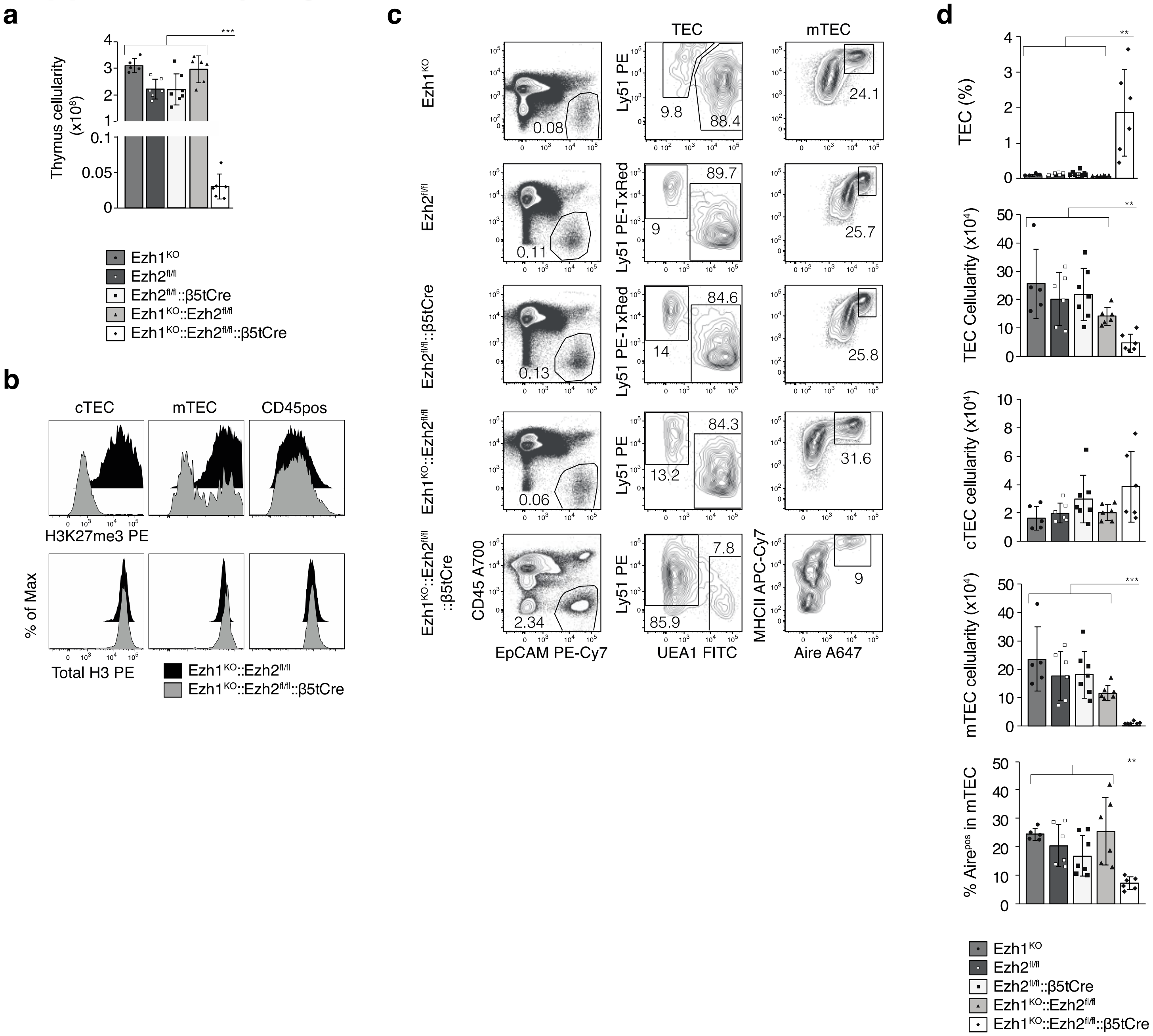

Supplementary Figure 2

Thymus phenotype of *Ezh1*^{KO}::*Ezh2*$^{fl/fl}$::*β5tCre* mice. (**a**) Thymus cellularity of mutant *Ezh1*^{KO}::*Ezh2*$^{fl/fl}$::*β5tCre* mice and *Ezh1*^{KO} , *Ezh2*$^{fl/fl}$, *Ezh2*$^{fl/fl}$::*β5tCre* and *Ezh1*^{KO}::*Ezh2*$^{fl/fl}$ control mice at 4 weeks of age. (**b**) Detection of H3K27me3 marks and histone H3 protein in cTEC, mTEC and thymocytes ($CD45^+$) isolated from *Ezh1*^{KO}::*Ezh2*$^{fl/fl}$::*β5tCre* (grey) and *Ezh1*^{KO}::*Ezh2*$^{fl/fl}$ (black) mice. (**c**) Representative contour plots showing frequencies of TEC (left panels), cTEC and mTEC distribution (middle panels) and Aire expressing mTEC (right panels). (**d**) Relative and absolute numbers of total TEC, relative and absolute cTEC and mTEC numbers and frequency of Aire expressing cells within mTEC. Histograms (**b**) are from one experiment representative of 2 independent experiments with n = 3 mice per group. Contour plots (**c**) are representative of data in bar graphs (**d**). Data in bar graphs (**a**, **d**) show the mean ± SD and are from one experiment (*Ezh1*^{KO}: n = 5, grey bars with black circles) or pooled from two independent experiments (*Ezh2*$^{fl/fl}$: n = 6, dark grey bars with white squares; *Ezh2*$^{fl/fl}$::*β5tCre*: n = 7, light grey bars with black squares; *Ezh1*^{KO}::*Ezh2*$^{fl/fl}$: n = 6, medium grey bars with black triangles; Ezh1^{KO}::Ezh2$^{fl/fl}$::β5tCre *Ezh2*$^{fl/fl}$: n = 6, white bars with black diamonds). n = biologically independent replicates per group. *p<0.05, **p<0.01, ***p<0.001 (two-tailed unpaired student's t test). Source data including exact statistical test values are provided as a source data file.

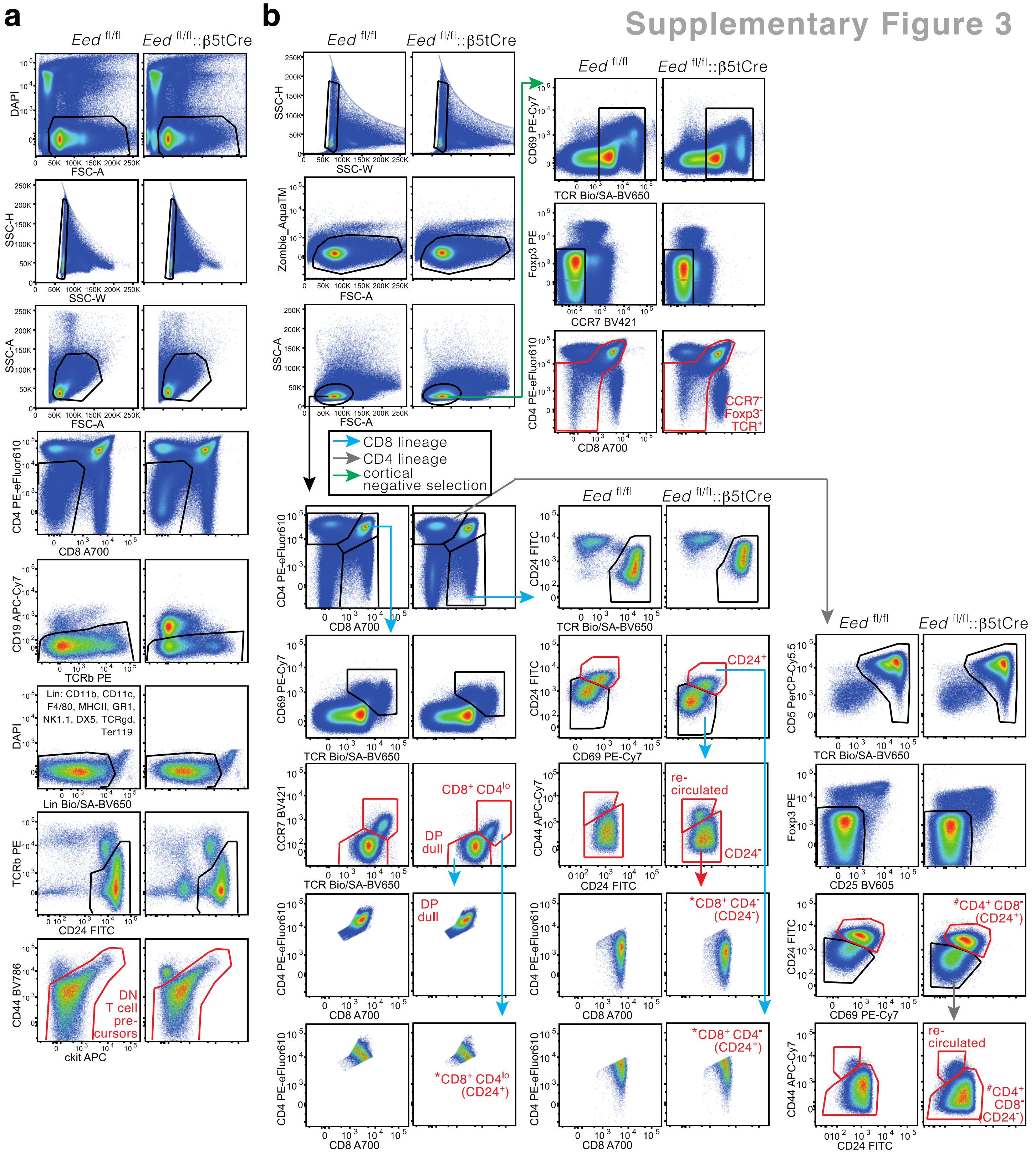

Supplementary Figure 3

Gating strategies used for definition of thymic subsets. (a) DN T cell precursors were defined as DAPI^{neg} singlets excluded from CD19^{pos}, Lin^{pos}, TCR^{pos}, CD24^{neg}, and CD44^{pos}ckit^{neg} cells. **(b)** After doublet and dead cell exclusion DP cells with upregulated TCR and CD69 expression were defined as positively selected DP^{dull} or CD8^{pos}CD4^{lo} according to CCR7 and TCR expression. TCR^{pos} CD8SP were divided into CD24^{pos} and CD24^{neg} subsets, re-circulated CD8 T cells were excluded from the latter. Populations marked with an asterix (*) were concatenated for all calculations concerning CD8 lineage selection. TCR^{pos} conventional CD4SP were defined as CD24^{pos} and CD24^{neg} subsets and re-circulated CD4 T cells were excluded from the latter. Populations marked with a hashtag (#) were concatenated for all calculations concerning CD4 lineage selection. In order to quantify negative selection of DP thymocytes in the cortex, Foxp3^{pos} and CCR7^{pos} cells together with SP subsets were excluded from TCR expressing thymocytes. Gates used for calculations and for display in Figures 3 and 4 are drawn in red.

Supplementary Figure 4

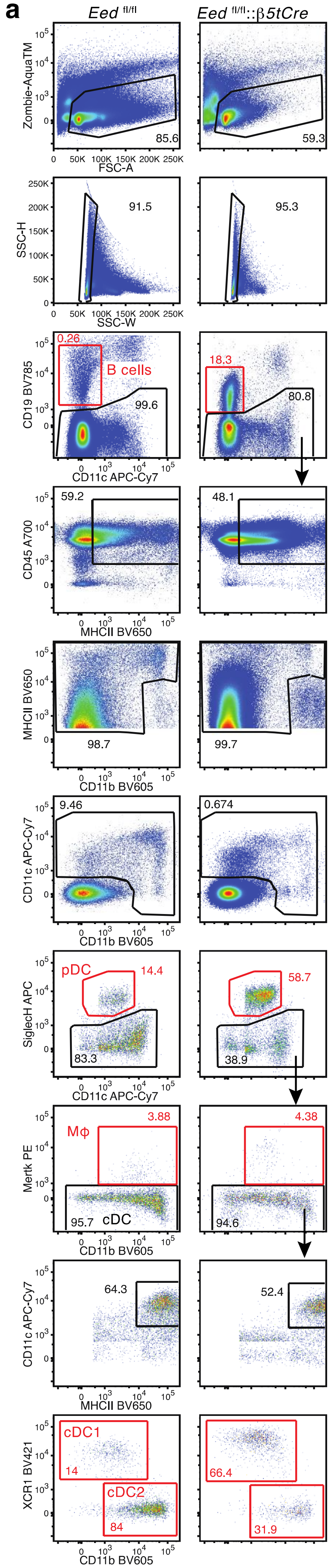

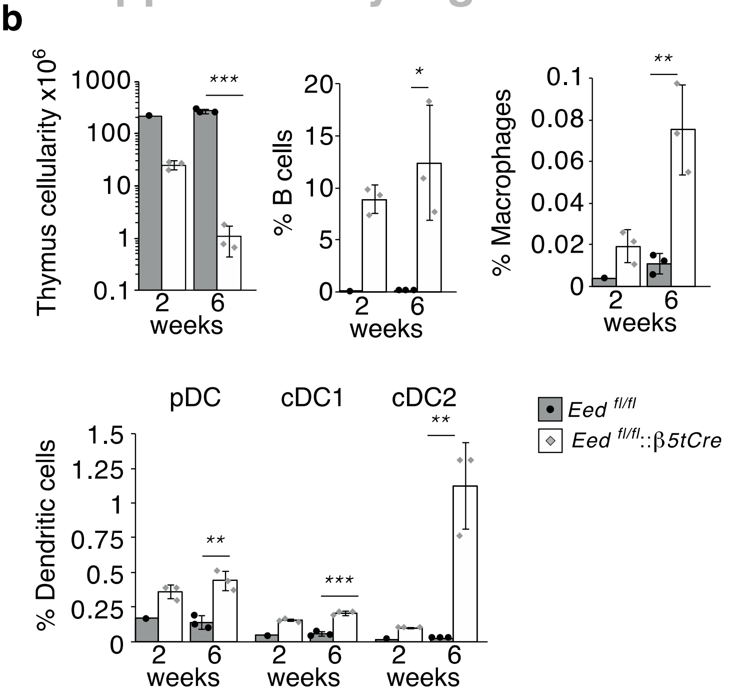

Supplementary Figure 4

Increased thymic frequencies of B cells, macrophages and DC in *Eed*$^{fl/fl}$::*β5tCre* mice. (**a**) Gating strategy to identify B cells, macrophages and DC subsets. Single cell suspensions were obtained by Collagenase D digestion. After B cell exclusion, MHCII expressing cells were cleared of eosinophils and CD11b^{neg} CD11c^{neg} cells. Plasmacytoid (p) DC were defined as SiglecH^{pos}, macrophages (Mø) as Mertk^{pos} and conventional (c) DC as MHCII^{pos}CD11c^{pos} cells. cDC1 and cDC2 were discriminated based on XCR1 and CD11b expression. Gates used for calculations are drawn in red. (**b**) Total thymus cellularity and the percentages of B cells, Mø , cDC of either type 1 (XCR1^{pos} CD11b^{low}) or 2 (XCR1^{neg} CD11b^{high}) and pDC (SiglecH^{pos} CD11c^{low}) at 2 weeks of age (*Eed*$^{fl/fl}$: *n* = 1, *Eed*$^{fl/fl}$::*β5tCre*: *n* = 3) and 6 weeks of age (*Eed*$^{fl/fl}$: *n* = 3, *Eed*$^{fl/fl}$::*β5tCre*: *n* = 3). *Eed* $^{fl/fl}$: grey bars with black circles. *Eed* $^{fl/fl}$::*β5tCre*: white bars with grey diamonds. *n* = biologically independent replicates per group. * $p < 0.05$, ** $p < 0.01$, *** $p < 0.001$ (two-tailed unpaired student's t test). Source data including exact statistical test values are provided as a source data file.

Supplementary Figure 5

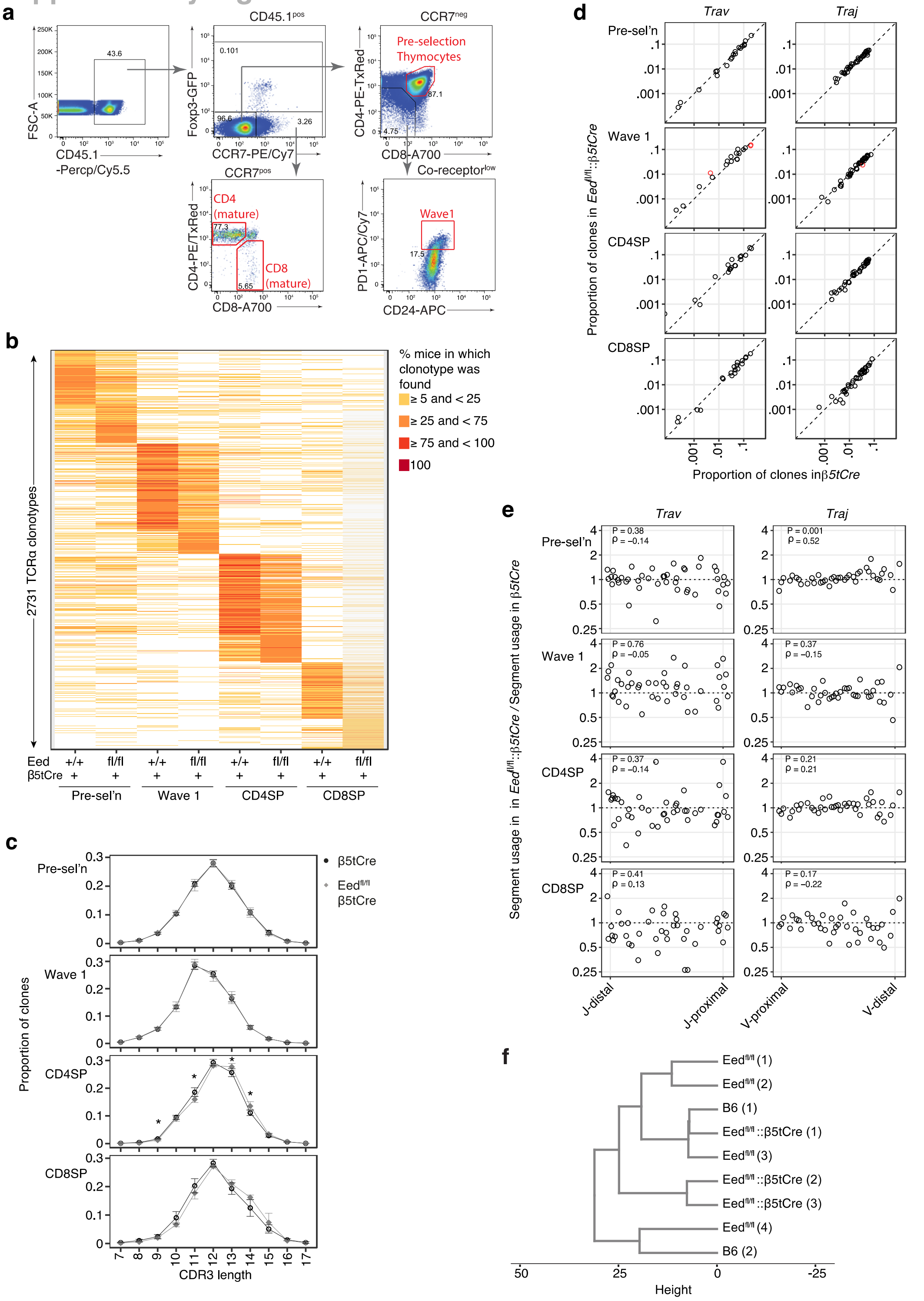

Supplementary Figure 5

TCR repertoire analysis of thymocytes in neonatal chimeras. (**a**) Gating strategy for thymocytes from *Eed*$^{fl/fl}$::*β5tCre* mice trans-planted with YAe62 TCRβ-tg bone marrow cells. Cells were first gated for CD45.1 positivity to identify cells of donor ($CD45.1^+$) origin. These cells were then gated for GFP-negativity to exclude T_{reg} (donor carry Foxp3-GFP transgene) and CCR7 expression. The $CCR7^-$ cells were then gated using CD4 and CD8 where the double positive cells are mainly pre-selection thymocytes. CD4 and CD8 co-receptor low cells were further gated for PD-1 positivity and isolated as Wave1 cells which are cortical thymocytes undergoing negative selection. The $CCR7^+$ cells were gated with CD4 and CD8 to isolate post selection mature CD4 and mature CD8. **(b-e)** Unless stated otherwise, comparisons were performed at the level of TCR catalogs, which were formed by aggregating samples of a given T cell subset/genotype combination. The *β5tCre* and *Eed*$^{fl/fl}$*::β5tCre* TCR catalogs contained the following numbers of samples: 9 and 14 mice, respectively (Pre-selection, Wave 1 and CD8SP); 8 and 14 mice, respectively (CD4SP). (**b**) Heatmap showing the incidence of individual TCRα clonotypes (*y*-axis) in TCR catalogs (*x*-axis). Within each TCR catalog, clonotypes were ranked based on the number of mice in which the clonotype was detected and then by the number of unique nucleotide sequences encoding each clonotype (referred to as "convergence"). Combining the top 500 clonotypes from each TCR catalog produced a list of 2731 unique clonotypes [which is fewer than 4000 (8 TCR catalogs × 500 clonotypes) because some clonotypes were in the top 500 of >1 TCR catalog]. Each clonotype was allocated to a "preferred" TCR catalog, defined as the TCR catalog in which the clonotype was detected in the greatest proportion of mice, with ties broken by allocation to the TCR catalog leftmost on the *x*-axis. Clonotypes were ordered on the *y*-axis from top to bottom based on "preferred" TCR catalog, as ordered from left to right on the *x*-axis. **(c)** TCRα CDR3 length distributions. For each sample, the proportion of TCRα clones with a CDR3 sequence of 7, 8, 9, 10, 11, 12, 13, 14, 15, 16 or 17 amino acids was determined. The CDR3 starts at the amino acid after the conserved cysteine at position 104 and ends at the amino acid before the conserved phenylalanine or tryptophan at position 118 in the IMGT numbering system. For each T cell subset (left), symbols show the mean, and error bars the SD, of *β5tCre* (black circles) and *Eed*$^{fl/fl}$*::β5tCre* (gray diamonds) samples. Samples with fewer than 100 clones were excluded. * indicates p = 0.03 using Mann Whitney tests (unpaired, two-sided, Bonferroni-corrected). **(d)** For each T cell subset (left), scatterplots show the mean proportion of clones using each *Trav* gene family (left column) or *Traj* gene segment (right column) in *Eed*$^{fl/fl}$::*β5tCre* samples (*y*-axis) versus control samples (*x*-axis). Red symbols indicate p = 0.02 (*Trav10*, increased in *Eed*$^{fl/fl}$*::β5tCre*), p = 0.03 (*Trav12*), p = 0.04 (*Trav7*) and p = 0.02 (*Traj45*) using Mann Whitney tests (unpaired, two-sided, Bonferroni-corrected). **(e)** Chromosomal positioning of *Trav* and *Trav* segment usage. After excluding clones that mapped to >1 *Trav* segment, the number of clones in each TCR catalog that used each *Trav* or *Traj* segment was determined and 1 was added to each value to avoid zeros in the dataset. The proportion of clones in each TCR catalog that used each *Trav* or *Traj* segment was determined. For each T cell subset (left), each symbol on the graphs represents the ratio of these proportions (*Eed*$^{fl/fl}$::*β5tCre*/control) on the *y*-axis as a function of the chromosomal position of the *Trav* (left column) or *Traj* (right column) segment on the *x*-axis, with the p and rho values of a two-sided Spearman's test for correlation on each graph. (**f**) Hierarchical clustering of the TCRα repertoire in thymocytes. TCRα repertoire from C57/Bl6 (n = 2), Eed$^{fl/fl}$ (n = 4), and Eed$^{fl/fl}$::β5tCre mice (n = 3) was determined by 5'RACE PCR and analyzed by hierarchical clustering using 8 parameters derived from the location of *Trav* and *Traj* elements within TCRα transcripts. Because of duplications and triplications of *Trav* gene elements, the analysis was restricted to unequivocally assigned upstream (distal) and downstream (proximal) *Trav* genes.

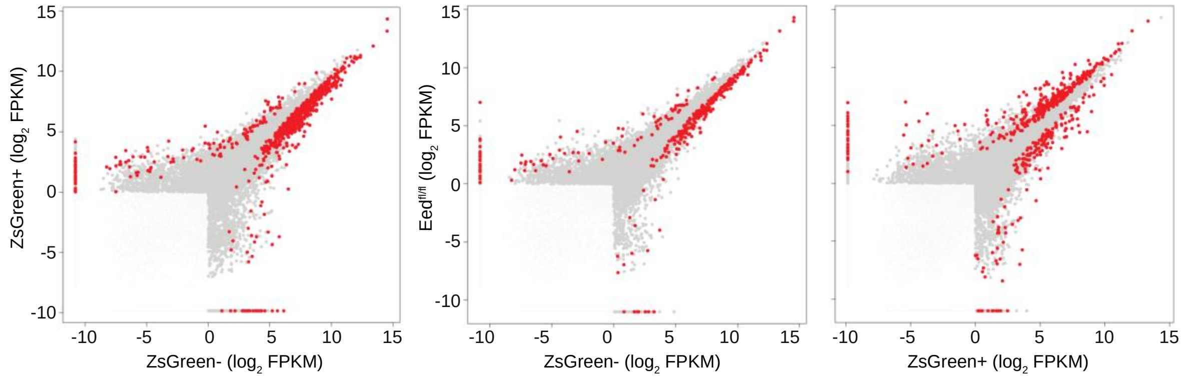

Supplementary Figure 6

Differential analysis between EED-deficient and EED-proficient mTEChi from *Eed*[fl/fl]::*ZsGreen*::*β5tCre* mice and mTEChi isolated from *Eed*[fl/fl]::*ZsGreen* mice. Scatter plots comparing $\log_2$ gene expression for each type of mTEC. Significant genes (FDR < 0.05) are highlighted in red. Genes with mean expression <1 FPKM in all types of mTEC were filtered out.

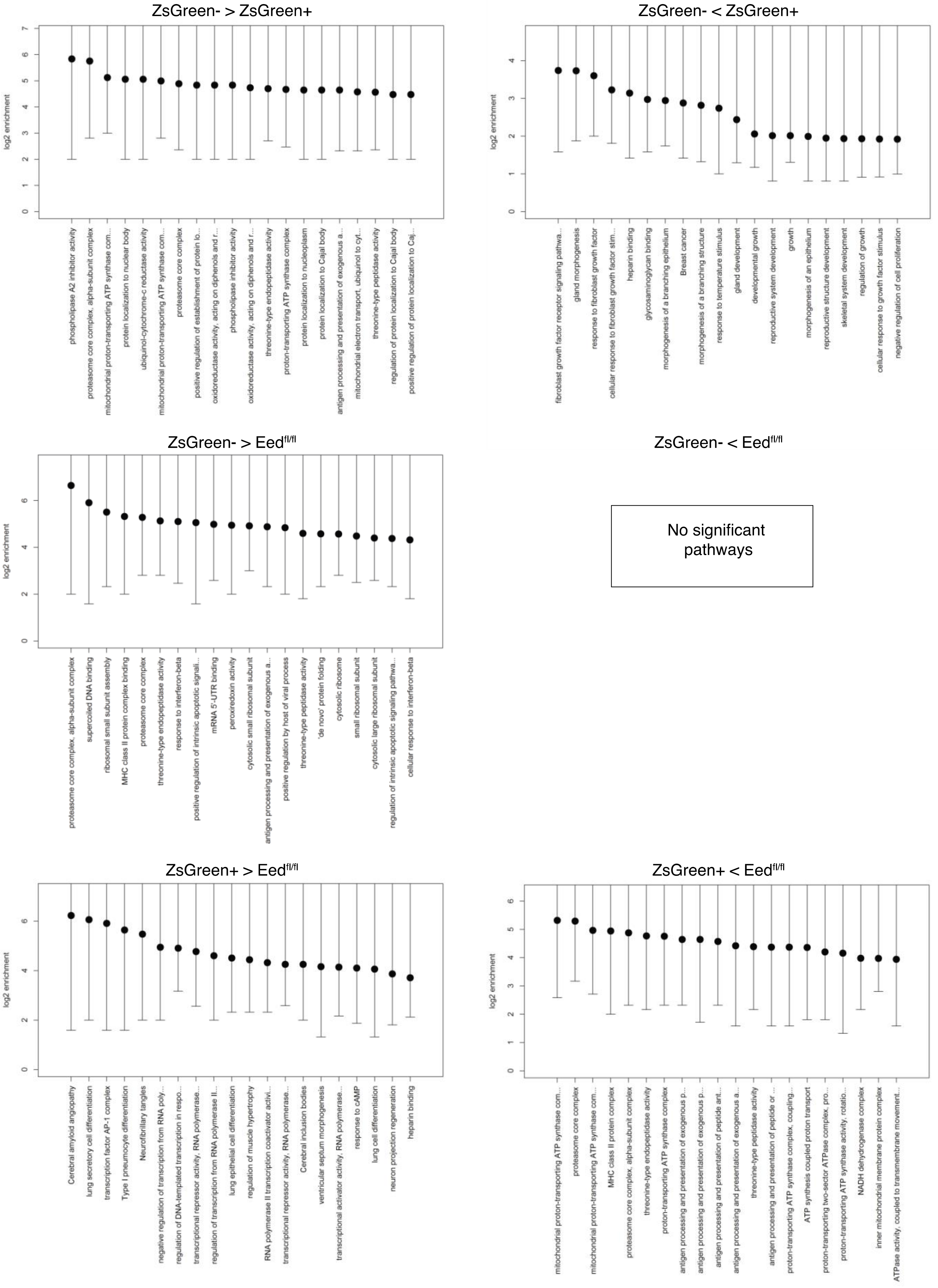

Supplementary Figure 7

Differential analysis between EED-deficient and EED-proficient mTEC^{hi} from *Eed*$^{fl/fl}$::*ZsGreen*::*β5tCre* mice and mTEC^{hi} isolated from *Eed*$^{fl/fl}$::*ZsGreen* mice. The top 20 significant gene ontology terms ranked by $\log_2$ enrichment for bulk mTEC^{hi} analysis. The points indicate $\log_2$ enrichment and the bars indicate 95% confidence intervals estimated from 1,000 permutations. Significance was calculated using one-tailed hypergeometric tests with correction for multiple hypothesis testing. Source data including exact statistical test values are provided as a source data file.

Supplementary Figure 8

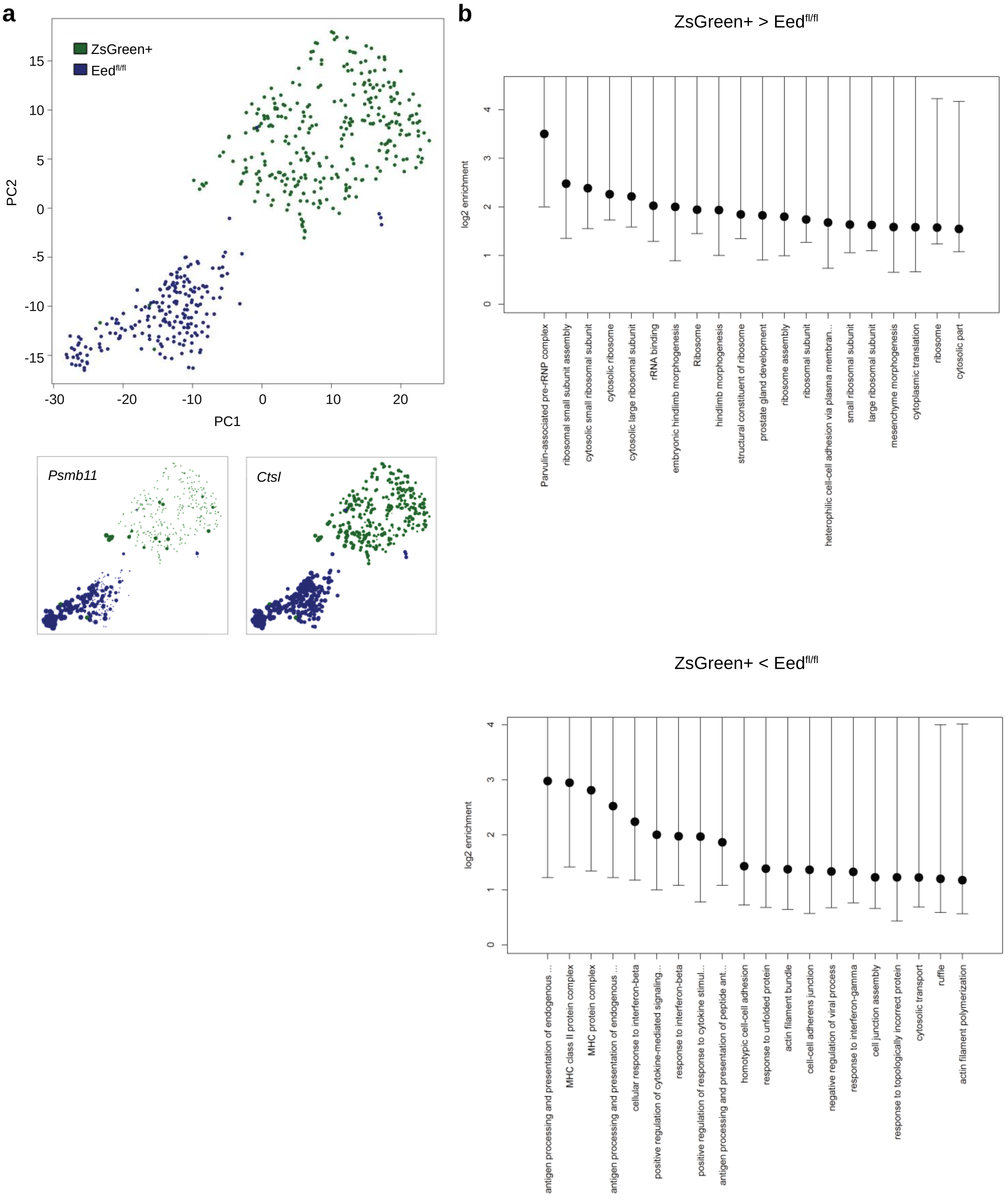

Supplementary Figure 8

Single cell analysis of cTEC. (**a**) tSNE clustering of single cTEC. Points are scaled by log_2 expression of *Psmb11* or *Ctsl* in the plots to the right. (**b**) The top 20 significant gene ontology terms ranked by log_2 enrichment for single cell cTEC analysis. The points indicate log_2 enrichment and the bars indicate 95% confidence intervals estimated from 1,000 permutations. Significance was calculated using one-tailed hypergeometric tests with correction for multiple hypothesis testing. Source data including exact statistical test values are provided as a source data file.

Supplementary Figure 9

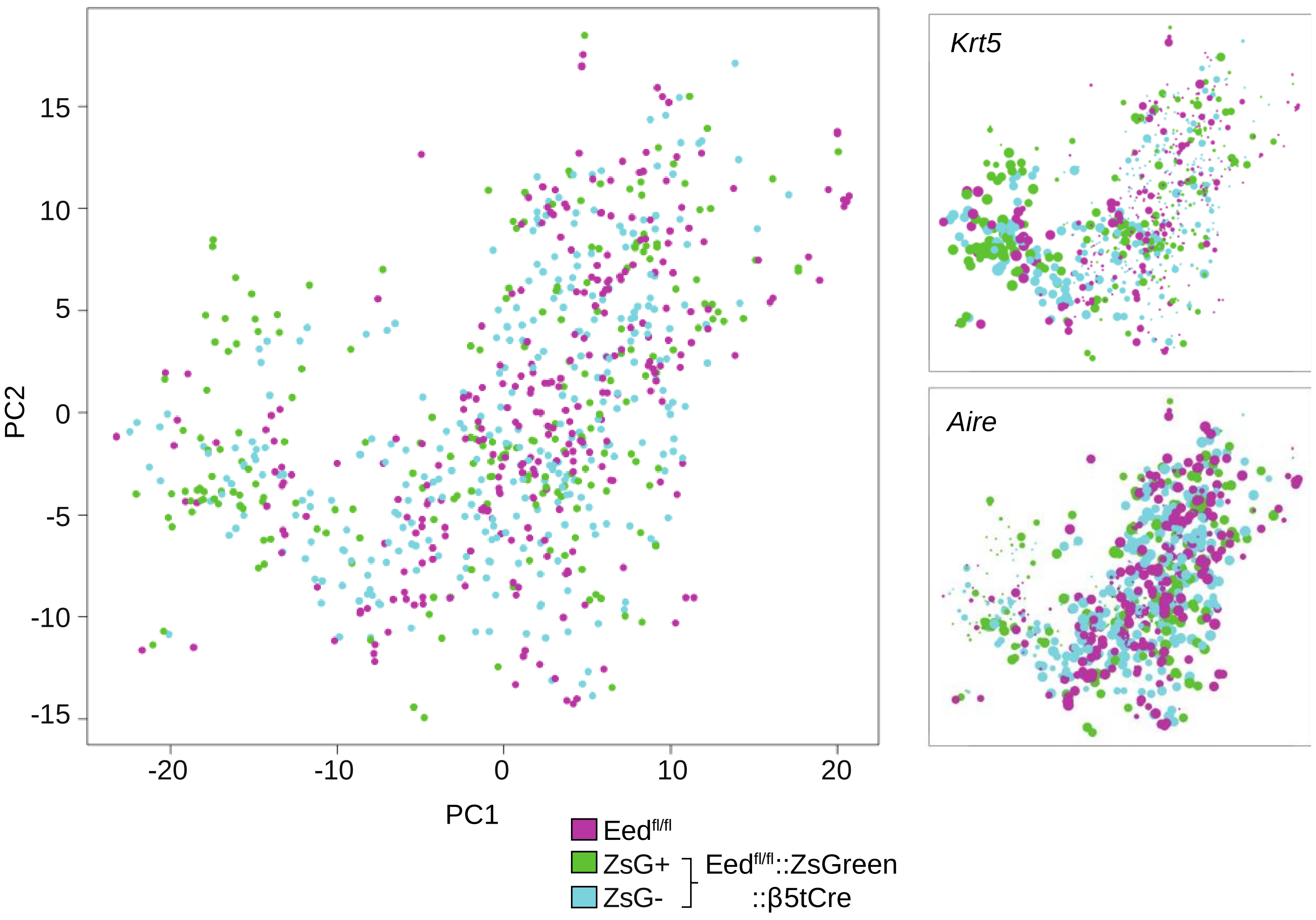

Supplementary Figure 9

Single cell analysis of mTEChi. tSNE clustering of single mTEC^{hi}. Points are scaled by $\log_2$ expression of *Krt5* (encoding Cytokeratin 5*)* or *Aire* in the plots to the right. *Eed* $^{fl/fl}$ (magenta), *Eed*$^{fl/fl}$*::ZsGreen::β5tCre*: ZsGreen-positive (green), ZsGreen-negative (blue).

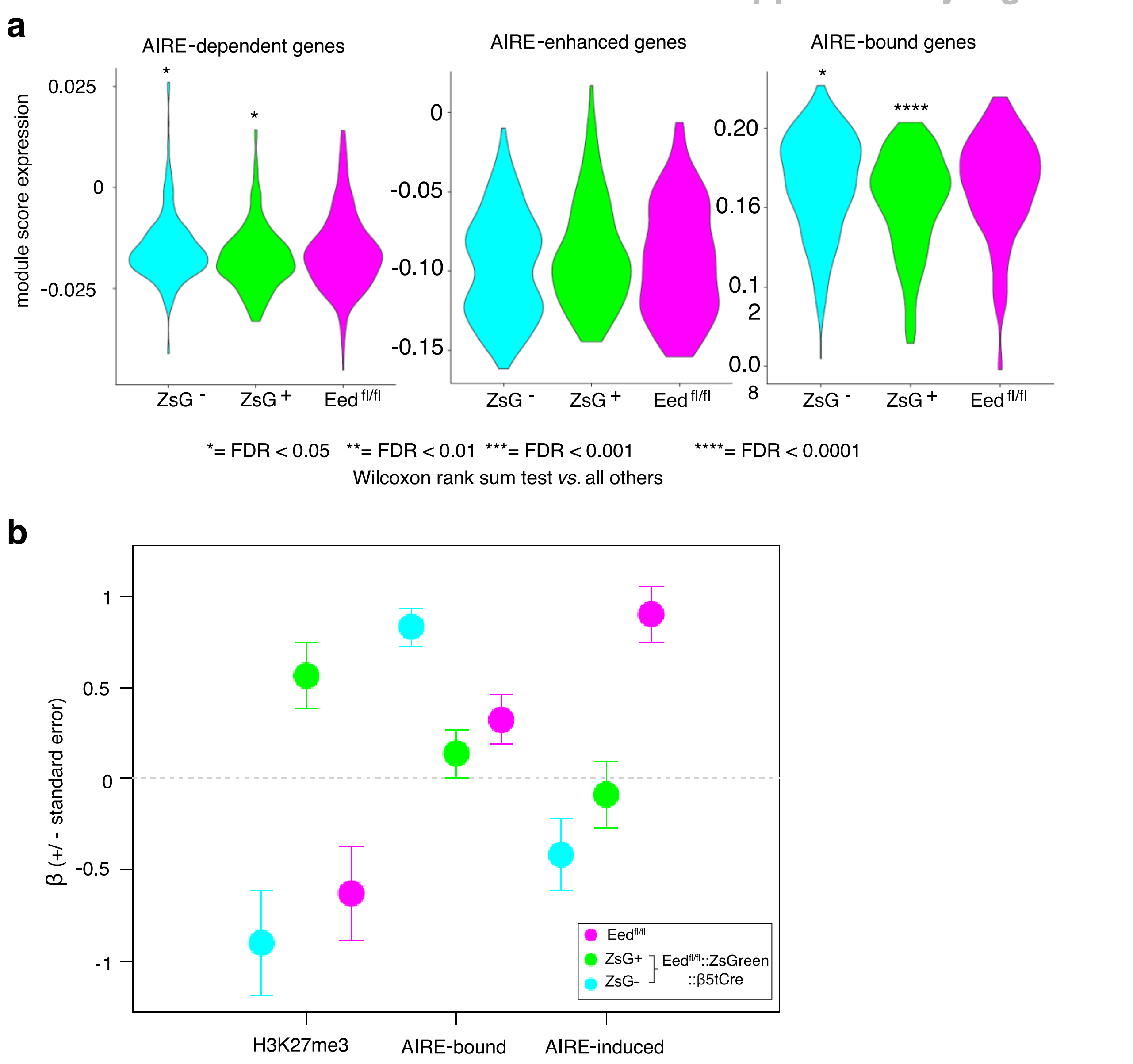

Supplementary Figure 10

AIRE-modulated expression in EED-deficient and -proficient mTECs. (**a**) Violin plots of gene module expression for AIRE-dependent, AIRE-enhanced or AIRE-bound genes. Significance was assessed by two-tailed Wilcoxon rank sum tests with Benjamini-Hochberg correction for multiple hypothesis testing. FDR = * < 0.05; ** < 0.01; *** < 0.001; and **** < 0.0001. (**b**) Multinomial regression weightings for H3K27me3-marked, AIRE-bound or AIRE-enhanced (AIRE-dependent plus AIRE-enhanced) gene sets for classifying cells as differentially expressed in the different mTEC types. Genes were classified as targets for H3K27me3 modification or AIRE if peaks fell within 5kb of their transcriptional start sites. Points show the regression coefficient (β) and bars show the standard error. *Eed* $^{fl/fl}$ (magenta), *Eed*$^{fl/fl}$*::ZsGreen::β5tCre*: ZsGreen-positive (green), ZsGreen-negative (blue). Source data including exact statistical test values are provided as a source data file.

Supplementary Figure 11

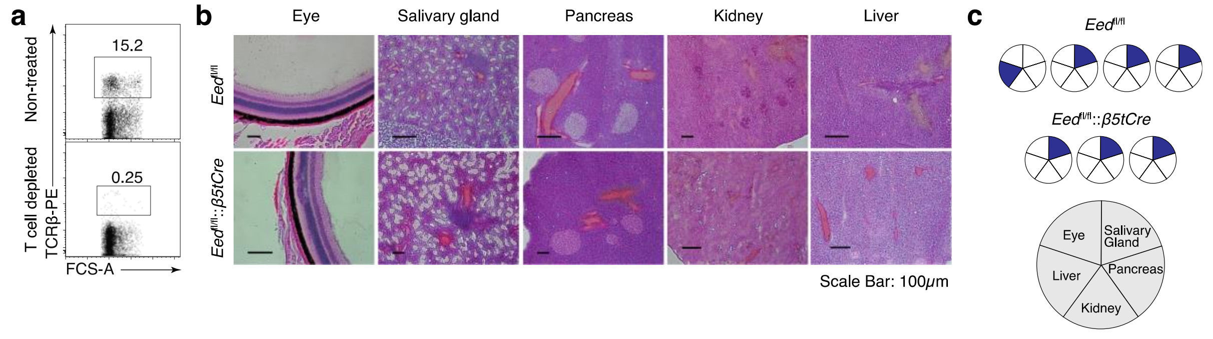

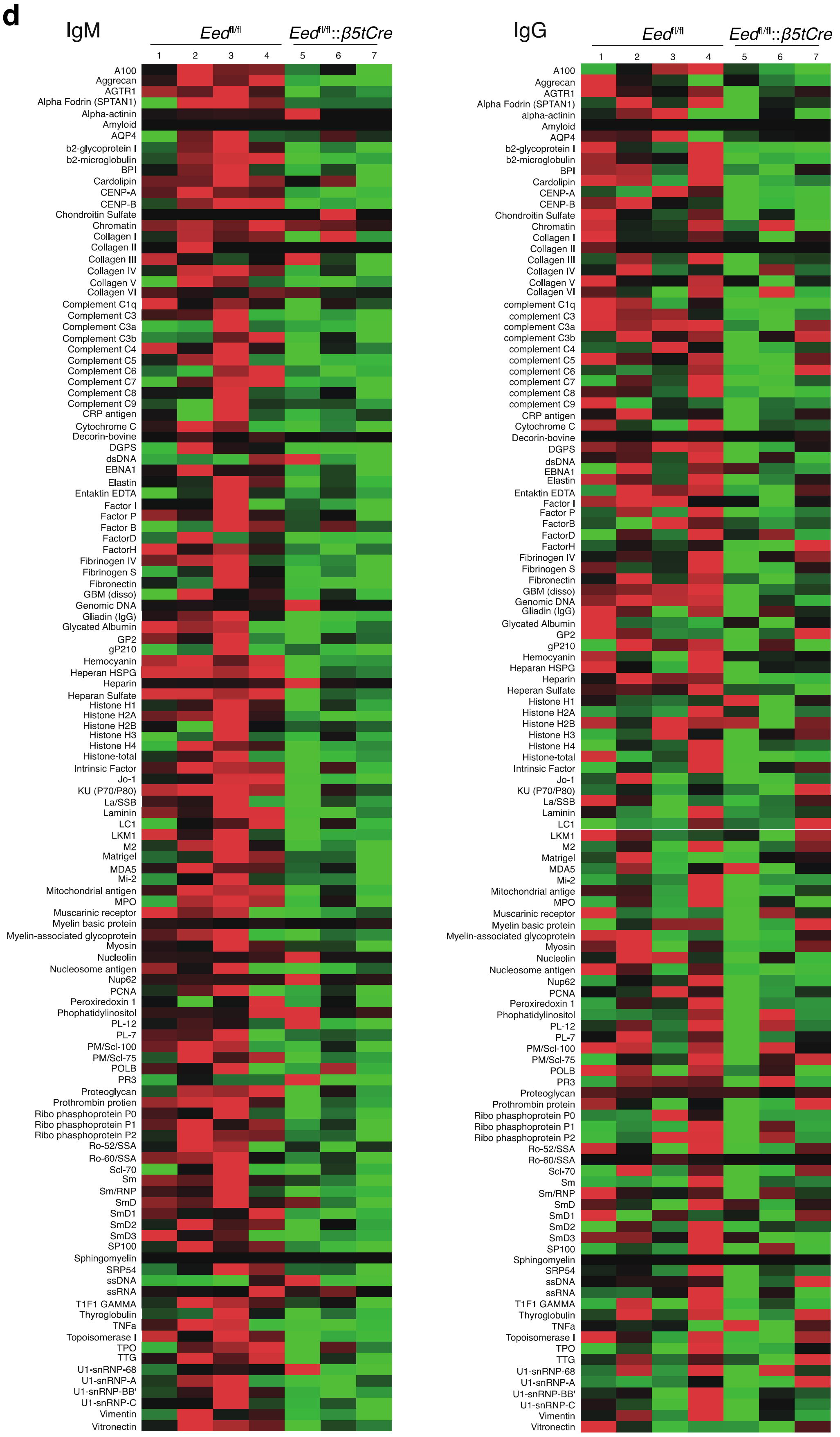

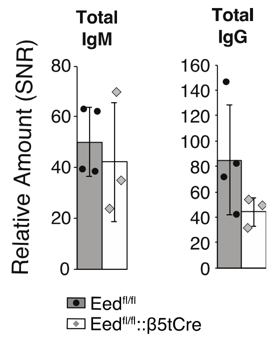

Supplementary Figure 11

Lack of autoimmunity in *Eed*$^{fl/fl}$::*β5tCre* mice. (**a**) Representative example of splenic T cell depletion 3 days after the last of 3 i.p. injections of CD4, CD8 and anti-Thy1.1 mAb. (**b**) Haematoxylin and Eosin tissue stains of indicated organs isolated from mice 20 weeks after T cell depletion using 3 i.p. doses of mAb cocktail. Stains are representative of at least 3 sections per tissue for each individual mouse. (**c**) Incidence of lymphocytic infiltrates in selected target tissues of *Eed*$^{fl/fl}$::*β5tCre* and *Eed*$^{fl/fl}$ mice. Coloured segments indicate histologically verified infiltration with organ key display on the bottom. (**d**) Self-antigen responsive IgM and IgG antibodies in serum of T cell-depleted mice. Left: Micro-array analysis of peripheral blood serum IgM and IgG from *Eed*$^{fl/fl}$::*β5tCre* and *Eed*$^{fl/fl}$ mice 20 weeks after T cell depletion at the age of 2 weeks using 3 i.p. injections of CD4, CD8 and anti-Thy1.1 mAb. Heat maps of antibody responsiveness to indicated antigens. Right: relative amount of serum IgM and IgG as signal to noise ratio (SNR). Bar graph shows mean with SD, *Eed*$^{fl/fl}$ (grey bars, black circles), *Eed*$^{fl/fl}$::*β5tCre* (white bars, grey diamonds. (**b, c, d**) female *Eed*$^{fl/fl}$ (n = 4), female *Eed*$^{fl/fl}$::*β5tCre* (n = 3). n = biologically independent replicates per group.

Supplementary Tables

Supplementary Table 1

Antibodies used for flow cytometry and immunohistology

Reactivity	Clone	Conjugation	Source	Dilution
Aire	5H12	eFluor660	eBioscience/ ThermoFisher	1:1000
CCR7	4B12	BV421, PE/Cy7	BioLegend	1:200
CD103	2E7	FITC	BioLegend	1:500
CD11b	M1/70	BV 605, Biotin	BioLegend	1:500
CD11c	N 418	APC/Cy7, Biotin	BioLegend	1:500
CD19	6D5	APC/Cy7	BioLegend	1:500
CD24	M1/69	FITC, PE, APC	BioLegend	1:1000
CD25	PC61	BV605, PerCP/Cy5.5	BioLegend	1:1000
CD3	145-2C11	Biotin	BioLegend	1:500
CD4	GK1.5	PE/Cy7, APC/Cy7	BioLegend	1:1000
CD4	RM4-5	PE/eFluor610	eBioscience/ ThermoFisher	1:1000
CD40	HM40-3	eFluor450	eBioscience/ ThermoFisher	1:200
CD44	IM7	BV785, FITC, PE/Cy7, APC-Cy7	BioLegend	1:1000
CD45	30-F11	AF 700	selfmade	1:500
CD45.1	A20	PE/Cy7, PE, PerCP/Cy5.5	BioLegend	1:500
CD5	53-7.3	PerCP/Cy5.5, APC	BioLegend	1:200
CD62L	MEL-14	FITC, PerCP/Cy5.5	BioLegend	1:500
CD69	H1.2F3	PE/Cy7, FITC	BioLegend	1:200
CD71	RI7217	PE/Cy7	BioLegend	1:200
CD8	53-6.7	AF 700	BioLegend	1:500
CD80	16-10A1	PerCP/Cy5.5	BioLegend	1:500
ckit	2B8	APC	BioLegend	1:200
Cytokeratin (CK) 14	Rabbit polyclonal	purified	BioLegend	1:1000
Cytokeratin (CK) 8	TROMA 1	Cy5, Biotin	selfmade	1:1000
DNA	DAPI	0.5 mg/ml	Sigma	1:10000
DX5	DX5	Biotin	BioLegend	1:500
EpCAM	G8.8	PerCP/Cy5.5, PE/Cy7, BV421	BioLegend	1:1000
EZH2	D2C9	AF647	Cell Signaling Technology	1:100
F4/80	A3-1	Biotin	BioLegend	1:2000
Foxp3	FJK-16s	PE, APC	eBioscience/ThermoFisher	5 ul/ test
Goat anti Rabbit IgG	polyclonal	AF647	Molecular Probes/Thermo Fisher	1:500
Gr-1	RB6-8C5	Biotin	BioLegend	1:500
H3K27me2	D18C8	AF647	Cell Signaling Technology	1:100
H3K27me3	C36B11	unconjugated, PE, AF647	Cell Signaling Technology	1:100
Helios	22F6	APC	BioLegend	5 ul/ test
Histone 3 (H3)	D1H2	unconjugated, PE	Cell Signaling Technology	1:100
ICOS	C398.4A	PE-Cy7	BioLegend	1:200
Lactadherin	--	FITC	Haematologic Technologies	1:100
Ly51	6C3	PE/Cy7, Biotin	BioLegend	1:500
MHC II	M5/114	APC/Cy7, BV650, PerCP/Cy5.5, Biotin	BioLegend	1:1000
NK1.1	PK 136	Biotin	BioLegend	1:500
PD-1	29F.1A12	BV785, APC/Cy7	BioLegend	1:200
Rabbit IgG control	DA1E	Unconjugated, AF647	Cell Signaling Technology	1:100
Sca1	D7	FITC, PE/Cy7, BV510	BioLegend	1:500
SiglecH	551	APC	BioLegend	1:500
Streptavidin	--	BV650, BV785, PerCP/Cy5.5		1:500
TCRb	H57-597	PE, Biotin	BioLegend	1:1000
TCRg	GL3	Biotin	BioLegend	1:500
Ter119	TER119	Biotin	BioLegend	1:200
TSPAN8	657909	PE	R&D Systems	1:100
UEA1	Lectin	Cy5, FITC, Biotin	Reactolab/Vector	1:500
XCR1	ZET	BV421	BioLegend	1:500

Supplementary Table 2

Summary of the mice, cells and TCR sequences analysed in each TCR catalog[a].

Host genotype	Subset[b]	# Mice[b]	# Cells (total)	# Cells $sample^{-1}$ (range)	Retained reads[b]	# Clones (total)	Clones per 100 cells	# Clonotypes[c] (total)
**$Eed^{fl/fl}$**	Pre-sel'n	9	450000	50000	148303	17573	3.9	15687
**$Eed^{fl/fl}$:: β5tCre**	Pre-sel'n	14	621000	10000-50000	215363	26555	4.3	22498
**$Eed^{fl/fl}$**	Wave 1	9	430000	30000-50000	156317	23044	5.4	17540
**$Eed^{fl/fl}$:: β5tCre**	Wave 1	14	211250	350-45000	193589	17624	8.3	13920
**$Eed^{fl/fl}$**	CD4SP	8	412000	25000-50000	171150	19895	4.8	14221
**$Eed^{fl/fl}$:: β5tCre**	CD4SP	14	262100	400-50000	180389	15685	6.0	10540
**$Eed^{fl/fl}$**	CD8SP	9	68800	1500-16000	89868	6538	9.5	5545
**$Eed^{fl/fl}$:: β5tCre**	CD8SP	14	46050	200-11000	77126	4050	8.8	3354

[a] Each row corresponds to 1 TCR catalog; [b] number of reads retained after reads detected <3 times in any given sample were excluded;

[c] a unique combination of *Trav* gene and CDR3 amino acid sequence was defined as a clonotype.

Supplementary Table 3

List of primers used.

Target	Sequence
Trav1	TCGTCGGCAGCGTCAGATGTGTATAAGAGACAGGCACATACAGCACCTCAG
Trav2*	TCGTCGGCAGCGTCAGATGTGTATAAGAGACAGACTCTGAGCCTGCCCT
Trav3.5D4.10	TCGTCGGCAGCGTCAGATGTGTATAAGAGACAGAACGACTCTCTCTGMACMTCACAG
Trav4	TCGTCGGCAGCGTCAGATGTGTATAAGAGACAGTCTGSTCTGAGATGCAATTTT
Trav5-1/5-4(D)	TCGTCGGCAGCGTCAGATGTGTATAAGAGACAGCTTCCYTTGGTATAAGCAAGA
Trav6-1/6-2	TCGTCGGCAGCGTCAGATGTGTATAAGAGACAGCAGATGCAAGGTCAAGTGAC
Trav6-3/6-4(D)	TCGTCGGCAGCGTCAGATGTGTATAAGAGACAGCAACTGCCAACAACAAGG
Trav6(D)-5	TCGTCGGCAGCGTCAGATGTGTATAAGAGACAGCTTCTCTGACTGTGAACTGTTC
Trav6-6	TCGTCGGCAGCGTCAGATGTGTATAAGAGACAGAGATTCCGTGACTCAAACAG
Trav6(D/N)-7	TCGTCGGCAGCGTCAGATGTGTATAAGAGACAGGCCTCAAGGGACAAAGAG
Trav7	TCGTCGGCAGCGTCAGATGTGTATAAGAGACAGCATGGCCTCTCTCAACTGCAC
Trav8	TCGTCGGCAGCGTCAGATGTGTATAAGAGACAGAGAGCCACCCTTGACAC
Trav9	TCGTCGGCAGCGTCAGATGTGTATAAGAGACAGGGCTTTGAGGCTGAGTT
Trav10	TCGTCGGCAGCGTCAGATGTGTATAAGAGACAGGGAGAGAAGGTCGAGCAAC
Trav11	TCGTCGGCAGCGTCAGATGTGTATAAGAGACAGAACAGGACACAGGCAAAG
Trav12	TCGTCGGCAGCGTCAGATGTGTATAAGAGACAGGGTTCCACGCCACTC
Trav13	TCGTCGGCAGCGTCAGATGTGTATAAGAGACAGTCCTTGGTTCTGCAGG
Trav14	TCGTCGGCAGCGTCAGATGTGTATAAGAGACAGGCAGCAGGTGAGACAAAG
Trav15	TCGTCGGCAGCGTCAGATGTGTATAAGAGACAGCTGSAYTGTTCATATRAGACAAGT
Trav16	TCGTCGGCAGCGTCAGATGTGTATAAGAGACAGATTATTCTCTGAACTTTCAGAAGC
Trav17	TCGTCGGCAGCGTCAGATGTGTATAAGAGACAGCAGTCCGTGGACCAGC
Trav18	TCGTCGGCAGCGTCAGATGTGTATAAGAGACAGTACTGGTACCGACAGGTC
Trav19	TCGTCGGCAGCGTCAGATGTGTATAAGAGACAGCAAGTTAAACAAAGCTCTCCATC
Trav21	TCGTCGGCAGCGTCAGATGTGTATAAGAGACAGGTGCACTTGCCTTGTAGC
Trac	GTCTCGTGGGCTCGGAGATGTGTATAAGAGACAGNNNNNNNNNNCAGGTTCTGGGTTCTGG
