# Supplementary material for: Indispensable epigenetic control of thymic epithelial cell development and function by polycomb repressive complex 2
Source: Nat Commun. 2021 Jun 24;12:3933. doi: 10.1038/s41467-021-24158-w (PMC8225857; doi:10.1038/s41467-021-24158-w)
Supplement: Supplementary file 2 — Reporting summary. [file 41467_2021_24158_MOESM2_ESM.pdf]

nature research

Corresponding author(s): Georg A. Holländer

Last updated by author(s): May 11, 2021

Reporting Summary

Nature Research wishes to improve the reproducibility of the work that we publish. This form provides structure for consistency and transparency in reporting. For further information on Nature Research policies, see our Editorial Policies and the Editorial Policy Checklist.

Statistics

For all statistical analyses, confirm that the following items are present in the figure legend, table legend, main text, or Methods section.

n/a	Confirmed	
☐	☒	The exact sample size (*n*) for each experimental group/condition, given as a discrete number and unit of measurement
☐	☒	A statement on whether measurements were taken from distinct samples or whether the same sample was measured repeatedly
☐	☒	The statistical test(s) used AND whether they are one- or two-sided<br>*Only common tests should be described solely by name; describe more complex techniques in the Methods section.*
☐	☒	A description of all covariates tested
☐	☒	A description of any assumptions or corrections, such as tests of normality and adjustment for multiple comparisons
☐	☒	A full description of the statistical parameters including central tendency (e.g. means) or other basic estimates (e.g. regression coefficient) AND variation (e.g. standard deviation) or associated estimates of uncertainty (e.g. confidence intervals)
☐	☒	For null hypothesis testing, the test statistic (e.g. *F*, *t*, *r*) with confidence intervals, effect sizes, degrees of freedom and *P* value noted<br>*Give P values as exact values whenever suitable.*
☒	☐	For Bayesian analysis, information on the choice of priors and Markov chain Monte Carlo settings
☒	☐	For hierarchical and complex designs, identification of the appropriate level for tests and full reporting of outcomes
☒	☐	Estimates of effect sizes (e.g. Cohen's *d*, Pearson's *r*), indicating how they were calculated

Our web collection on statistics for biologists contains articles on many of the points above.

Software and code

Policy information about availability of computer code

Data collection	FACSDiva (BD Biosciences v 8.0.1)
Data analysis	Flowjo (Treestar Inc. v 9.8.3 v 10.7.1), Excel (Microsoft v 16.16.20), FactomineR (v 2.4), Factoextra (v 1.0.7), EstimateS (v 9.1.0), LymAnalyzer (v 1.2.2), iNEXT (v 2.0.20), MIGEC (v 1.2.1), HISAT (v 0.1.6), Trimmomatic (v 0.37), FastQC (v 0.11.6), HTSeq (v 0.7.1), edgeR (v 3.6.8), gProfiler (v 1741), SCnorm (v 0.99.5), linnorm (v 2.0.8), scDD (v 1.4.0), BWA (v 0.5.9), Stampy (v 1.0.28), MACS2 (v 2.0.10.20131028), GENIE3 (v 1.10.0), RcisTarget (v 1.8.0)

For manuscripts utilizing custom algorithms or software that are central to the research but not yet described in published literature, software must be made available to editors and reviewers. We strongly encourage code deposition in a community repository (e.g. GitHub). See the Nature Research guidelines for submitting code & software for further information.

Data

Policy information about availability of data

All manuscripts must include a data availability statement. This statement should provide the following information, where applicable:
- Accession codes, unique identifiers, or web links for publicly available datasets
- A list of figures that have associated raw data
- A description of any restrictions on data availability

The full TCR sequence dataset is available at the Sequence Read Archive (BioProject ID PRJNA565651 [https://www.ncbi.nlm.nih.gov/bioproject/PRJNA565651]) and processed data is available from the authors on request. RNA-seq and ChIP-seq sequencing data is available at the Gene Expression Omnibus (GSE112050 [https://www.ncbi.nlm.nih.gov/geo/query/acc.cgi?acc=GSE112050] and GSE114713 [https://www.ncbi.nlm.nih.gov/geo/query/acc.cgi?acc=GSE114713]). AIRE ChIP-seq data was downloaded from GSE92597 [https://www.ncbi.nlm.nih.gov/geo/query/acc.cgi?acc=GSE92597]. Source data for Figures 1, 2, 3, 4, 6, 7, 9, 10 and Supplementary Figures 2, 4, 7, 8, 10, 11 are provided with the paper. Other data that support the findings of this study are available from the corresponding author

upon reasonable request.

Field-specific reporting

Please select the one below that is the best fit for your research. If you are not sure, read the appropriate sections before making your selection.

- [x] Life sciences
- [ ] Behavioural & social sciences
- [ ] Ecological, evolutionary & environmental sciences

For a reference copy of the document with all sections, see nature.com/documents/nr-reporting-summary-flat.pdf

Life sciences study design

All studies must disclose on these points even when the disclosure is negative.

Sample size	The sample size used and estimates of variation within groups were based on published results using similar approaches.https://www.nature.com/articles/ni.3537 https://www.nature.com/articles/ni.2869
Data exclusions	No data points were excluded
Replication	Experiments were reliably repeated at least two times and/or with sufficient animals per group to demonstrate statistical significance
Randomization	Whenever possible, age and sex matched littermates were used according to their genotype, otherwise, no randomization was used as this is not relevant to the field of study.
Blinding	Due to logistical reasons investigators were not blinded to experimental group allocations since data analysis was strictly quantitative and not subjective

Reporting for specific materials, systems and methods

We require information from authors about some types of materials, experimental systems and methods used in many studies. Here, indicate whether each material, system or method listed is relevant to your study. If you are not sure if a list item applies to your research, read the appropriate section before selecting a response.

Materials & experimental systems

n/a	Involved in the study	
☐	☒	Antibodies
☒	☐	Eukaryotic cell lines
☒	☐	Palaeontology and archaeology
☐	☒	Animals and other organisms
☒	☐	Human research participants
☒	☐	Clinical data
☒	☐	Dual use research of concern

Methods

n/a	Involved in the study	
☐	☒	ChIP-seq
☐	☒	Flow cytometry
☒	☐	MRI-based neuroimaging

Antibodies

Antibodies used

Reactivity	Clone	Conjugation	Source	Dilution	Catalog Nr
Aire	5H12	eFluor660	eBioscience/ ThermoFisher	1:1000	50-5394-82
CCR7	4B12	BV421	BioLegend	1:200	120120
		PE/Cy7			1120124
CD103	2E7	FITC	BioLegend	1:500	121420
CD11b	M1/70	BV 605	BioLegend	1:500	101237
		Biotin			101204
CD11c	N 418	APC/Cy7	BioLegend	1:500	117324
		Biotin			117304
CD19	6D5	APC/Cy7	BioLegend	1:500	115530
CD24	M1/69	FITC	BioLegend	1:1000	101806
		PE			101808
		APC			101814
CD25	PC61	BV605	BioLegend	1:1000	102036
		PerCP/Cy5.5			102029
CD3	145-2C11	Biotin	BioLegend	1:500	100304
CD4	GK1.5	APC/Cy7	Biolegend	1:1000	100414
		PE/Cy7			100422
	RM4-5	PE/eFluor610	eBioscience/ ThermoFisher		61-0042-82

CD40	HM40-3	eFluor450	eBioscience/ ThermoFisher	1:200	48-0402-82
CD44	IM7	BV785	BioLegend	1:1000	103059
		FITC			103006
		PE/Cy7			103030
		APC-Cy7			103028
CD45	30-F11	AF 700	selfmade	1:500	
CD45.1	A20	PE/Cy7	BioLegend	1:500	101730
		PE			110708
		PerCP/Cy5.5			101728
CD5	53-7.3	PerCP/Cy5.5	BioLegend	1:200	100624
		APC			100626
CD62L	MEL-14	FITC	BioLegend	1:500	104406
		PerCP/Cy5.5			104432
CD69	H1.2F3	PE/Cy7	BioLegend	1:200	104512
		FITC			104506
CD71	RI7217	PE/Cy7	BioLegend	1:200	113812
CD8	53-6.7	AF 700	BioLegend	1:500	100730
CD80	16-10A1	PerCP/Cy5.5	BioLegend	1:500	104722
ckit	2B8	APC	BioLegend	1:200	105812
Cytokeratin (CK) 14	Rabbit polyclonal	purified	BioLegend	1:1000	905301
Cytokeratin (CK) 8	TROMA 1	Cy5	Self-made	1:1000	
		Biotin	Self-made		
DNA	DAPI 0.5 mg/ml		Sigma	1:10000	D1306
DX5	DX5	Biotin	BioLegend	1:500	108904
EpCAM	G8.8	PerCP/Cy5.5	BioLegend	1:1000	118220
		PE/Cy7			118206
		BV421			118225
EZH2	D2C9	AF647	Cell Signaling Technology	1:100	45638
F4/80	A3-1	Biotin	BioLegend	1:2000	123106
Foxp3	FJK-16s	PE	eBioscience/ThermoFisher	5 ul/ test	12-5773-82
		APC			17-5773-82
Goat anti Rabbit IgG	polyclonal	AF647	Molecular Probes/ThermoFisher	1:500	A21244
Gr-1	RB6-8C5	Biotin	BioLegend 1:500 108404		
H3K27me2	D18C8	AF647	Cell Signaling Technology	1:100	12244
H3K27me3	C36B11	Unconjugated	Cell Signaling Technology	1:100	9733
		PE			40724
		AF647			12158
Helios	22F6	APC	BioLegend	5 ul/ test	137222
Histone 3 (H3)	D1H2	none	Cell Signaling Technology	1:100	4499
		PE			82241
		AF647			12230
ICOS	C398.4A	PE-Cy7	BioLegend	1:200	313520
Lactadherin	--	FITC	Haematologic Technologies	1:100	JJ1114-1ML
Ly51	6C3	PE/Cy7	BioLegend	1:500	108314
		Biotin			108304
MHC II	M5/114	APC/Cy7	Biolegend	1:1000	107628
		BV650			107641
		PerCP/Cy5.5			107626
		Biotin			107604
NK1.1	PK 136	Biotin	BioLegend	1:500	108704
PD-1	29F.1A12	BV785	BioLegend	1:200	135225
		APC/Cy7			135224
Rabbit IgG control	DA1E	Unconjugated	Cell Signaling Technology	1:100	3900
		AF647			2985
Sca1	D7	FITC	BioLegend	1:500	108106
		PE/Cy7			108114
		BV510			108129
SiglecH	551	APC	BioLegend	1:500	129612
Streptavidin	--	BV650	BioLegend	1:500	405232
		BV785			405249
		PerCP/Cy5.5			405214
TCRb	H57-597	PE	BioLegend	1:1000	109208
		Biotin			109204
TCRg	GL3	Biotin	BioLegend	1:500	118103
Ter119	TER119	Biotin	BioLegend	1:200	116204
TSPAN8	657909	PE	R&D Systems	1:100	FAB6524P
UEA1	--	FITC	Reactolab/Vector	1:500	F.L1061
		Cy5	self-conjugated		L-1060

XCR1	ZET	Biotin<br>BV421	self conjugated<br>BioLegend		1:500	L-1060<br>148216

Validation

All antibodies came from commercial vendors and were validated by the manufacturers on their official websites

Animals and other organisms

Policy information about studies involving animals; ARRIVE guidelines recommended for reporting animal research

Laboratory animals

C57BL/6, β5t-Cre, Eedfl/fl, Eedfl/wt, Eedfl/wt::β5tCre, Eedfl/fl::β5tCre, Eedfl/fl::ZsGreen, Eedfl/fl::ZsGreen::β5t-Cre, Ezh1-/-, Ezh2fl:fl, Ezh2fl/fl::β5tCre, Ezh1-/-::Ezh2fl/fl, Ezh1-/-::Ezh2fl/fl::β5tCre, YAe62β, C57BL/6 Rag2-/-, B6.Cg-Gt(ROSA)26Sortm6(CAG-ZsGreen1) Hze/J

Wild animals

The study did not involve wild animals

Field-collected samples

The study did not involve samples collected in the field

Ethics oversight

The study was approved by the veterinary cantonal authority of Basel Stadt

Note that full information on the approval of the study protocol must also be provided in the manuscript.

ChIP-seq

Data deposition

- [x] Confirm that both raw and final processed data have been deposited in a public database such as GEO.
- [x] Confirm that you have deposited or provided access to graph files (e.g. BED files) for the called peaks.

Data access links
May remain private before publication.

https://www.ncbi.nlm.nih.gov/geo/query/acc.cgi?acc=GSE112050

Files in database submission

mTEChi_input-1
mTEChi_input-2
mTEChi_input-3
mTEChi_input-4
mTEChi_input-5
mTEChi_K27me3-1
mTEChi_K27me3-2

Genome browser session
(e.g. UCSC)

The following tracks can be loaded into UCSC for visualisation
track type=bigBed name=TEC_histone_MACS2_peaks_IDR_lt_1pc description=TEC_histone_MACS2_peaks_IDR_lt_1pc visibility=1 itemRgb=On db=mm10 bigDataUrl=https://sara.molbiol.ox.ac.uk/public/ahandel/ TEC_histone_marks_IDR_0.01_merged_vs_input_merged_IDR_0.01_MAPQ_10_minus_blacklist.bb
track type=bigBed name=TEC_histone_MACS2_peaks_FDR_lt_1pc description=TEC_histone_MACS2_peaks_FDR_lt_1pc visibility=0 itemRgb=On db=mm10 bigDataUrl=https://sara.molbiol.ox.ac.uk/public/ahandel/ TEC_histone_marks_mapq_10.bb
track type=bigWig name=mTEChi_H3K27me3 description=mTEChi_H3K27me3 visibility=2 color=0,255,0 db=mm10 windowingFunction=mean smoothingWindow=3 bigDataUrl=https://sara.molbiol.ox.ac.uk/public/ahandel/ mTEChi_K27me3-1_merged_signal_mapq_10_FE.bw

Methodology

Replicates

5 input samples, 2 ChIP samples

Sequencing depth

sampleID totalReads uniquelyMapped readLength seqType
mTEChi_input-1 124684134 81972428 100 paired
mTEChi_input-2 171484747 132002358 100 paired
mTEChi_input-3 149584015 116723420 100 paired
mTEChi_input-4 155359183 118675756 100 paired
mTEChi_input-5 180485396 136952242 100 paired
mTEChi_K27me3-1 89537576 67802032 100 paired
mTEChi_K27me3-2 87695181 67117440 100 paired

Antibodies

anti-H3K27me3 (07-449, millipore)

Peak calling parameters

BWA (version 0.7.12) was used for pre-alignment of 100 basepair paired-end reads against the UCSC mm10 genome with the arguments “bwa aln -t8 -q10 <forward/reverse reads>” and “bwa sampe <forward sai> <reverse sai>”. Pre-aligned bam files were further aligned with Stampy (version 1.0.23) with the arguments “-t 8 –process-part=n/10 –bamkeepgoodreads”. Reads were filtered to obtain concordantly mapping read pairs with a MAPQ score > 10. Picard Tools was used to remove duplicate fragments. Peaks were called for broad marks (H3K9me3 and H3K27me3) using MACS2 (version 2.0.10.20131028) with the arguments “–keep-dup all –

	broad."
Data quality	We used FastQC to assess read quality and Trimmomatic to remove adapter sequences (transposons or their reverse complement), trim the first and last 3 bases of each read based on sequencing quality, trim sequences based on a sliding window (4:15), and retain reads with a minimum length of 20 bases. Irreproducibility discovery rate were estimated for peaks as detailed in Li Q, Brown JB, Huang H, Bickel PJ. Measuring reproducibility of high-throughput experiments. Ann Appl Stat. (2011) 5:1752–79. doi: 10.1214/11-AOAS466 and Kundaje A. ENCODE: TF ChIP-seq Peak Calling Using the Irreproducibility Discovery Rate (IDR) Framework. Available online at: https://sites.google.com/site/anshulkundaje/projects/idr (Accessed March 22, 2014). Peaks were filtered against the ENCODE mm10 blacklist.
Software	Trimmomatic (v 0.37), FastQC (v 0.11.6), BWA (v 0.5.9), Stampy (v 1.0.28), MACS2 (v 2.0.10.20131028)

Flow Cytometry

Plots

Confirm that:

- [x] The axis labels state the marker and fluorochrome used (e.g. CD4-FITC).
- [x] The axis scales are clearly visible. Include numbers along axes only for bottom left plot of group (a 'group' is an analysis of identical markers).
- [x] All plots are contour plots with outliers or pseudocolor plots.
- [x] A numerical value for number of cells or percentage (with statistics) is provided.

Methodology

Sample preparation	Thymic epithelial cell isolation<br>Thymic lobes were cleaned from fatty tissues and incubated with Liberase and DNaseI (Roche Diagnostics; 200 µg/ml and 30 µg/ml, respectively; 45 min, 37ºC) to obtain a cell suspension which was subsequently filtered through a nylon mesh (100micro pore size, Sefar Nitex) to remove debris. With the exception of Figure 1d, wild type TEC were magnetically enriched using the AutoMACS Pro Separator (Miltenyi Biotec) to obtain sufficient cells for analysis.<br><br>Thymic macrophage and dendritic cell isolation<br>Cleaned thymic lobes were incubated with Collagenase D and DNaseI (Roche Diagnostics; 1 mg/ml and 30 µg/ml, respectively; 45 min, 37ºC) to obtain a cell suspension which was subsequently filtered through a nylon mesh (100 µm pore size, Sefar Nitex) to remove debris and stained.<br><br>Thymocyte and peripheral T cell isolation<br>Cleaned organs were gently squeezed with bent tweezers in between a nylon mesh (100 µm pore size, Sefar Nitex) to obtain a single cell suspension.
Instrument	BD FACSAria II, BD LSR Fortessa
Software	FACSDiva (BD Biosciences v 8.0.1), Flowjo (Treestar Inc. v 9.8.3 v 10.7.1)
Cell population abundance	Sorted T cells for functional assays were re-analysed directly after sorting and sort purity was > 95%. Other sorted cell populations were not re-analysed due to very low abundance.
Gating strategy	All gating strategies are provided as supplementary information

- [x] Tick this box to confirm that a figure exemplifying the gating strategy is provided in the Supplementary Information.
