# Supplementary material for: Indispensable epigenetic control of thymic epithelial cell development and function by polycomb repressive complex 2
Source: Nat Commun. 2021 Jun 24;12:3933. doi: 10.1038/s41467-021-24158-w (PMC8225857; doi:10.1038/s41467-021-24158-w)
Supplement: Supplementary file 3 — Description of Additional Supplementary Files. [file 41467_2021_24158_MOESM3_ESM.pdf]

Description of Additional Supplementary Files

File Name: Supplementary Data 1
Description: Eed_ZsGneg mTEChi vs. Eed_ZsGpos mTEChi differentially expressed genes

File Name: Supplementary Data 2
Description: Eed_ZsGneg mTEChi vs. control mTEChi differentially expressed genes

File Name: Supplementary Data 3
Description: Eed_ZsGpos mTEChi vs. control mTEChi differentially expressed genes
